# Effect of tracheal antimicrobial peptide on the development of *Mannheimia haemolytica* pneumonia in cattle

**DOI:** 10.1371/journal.pone.0225533

**Published:** 2019-11-26

**Authors:** Ksenia Vulikh, Laura L. Bassel, Lauren Sergejewich, Emily I. Kaufman, Joanne Hewson, Janet I. MacInnes, Saeid Tabatabaei, Jeff L. Caswell

**Affiliations:** 1 Department of Pathobiology, University of Guelph, Guelph, Ontario, Canada; 2 Department of Clinical Studies, University of Guelph, Guelph, Ontario, Canada; Faculte de medecine veterinaire, Universite de Montreal, CANADA

## Abstract

Bacterial pneumonia causes significant economic loss to the beef industry and occurs at times of stress and viral infection. Administering antibiotics to at-risk calves is often used to prevent the disease, but alternatives to mass treatment with antibiotics are needed. Tracheal antimicrobial peptide (TAP), a β-defensin naturally produced by bovine airways, has bactericidal activity against the pathogens that cause pneumonia in cattle. However, TAP expression is suppressed by glucocorticoid (stress) and viral infection. We hypothesized that delivering TAP to the respiratory tract would prevent development of pneumonia in calves infected with *Mannheimia haemolytica*. Clean-catch calves (i.e. obtained prior to contact with the dam) were challenged by aerosol with *M*. *haemolytica*, and TAP or water was delivered to the respiratory tract at 0.3, 2 and 6 hours post-infection. TAP treatment did not protect against development of disease. Calves treated with TAP had similar bacterial loads in the nasal cavity and lung compared to calves treated with water. Similarly, TAP treatment did not affect the development of clinical signs, elevated rectal temperatures, or increased levels of blood neutrophils, haptoglobin and fibrinogen that occurred after bacterial challenge. Postmortem gross and histologic lung lesions were also similar in the two groups. To determine why there was a lack of protective effect, we tested the effect of substances in respiratory lining fluid on the bactericidal activity of TAP. Physiologic concentrations of sodium chloride inhibited TAP bactericidal activity in vitro, as did serum at concentrations of 0.62 to 2.5%, but concentrated bronchoalveolar lavage fluid had no consistent effect. These findings suggest that TAP does not have in vivo bactericidal activity against *M*. *haemolytica* because of interference by physiological sodium chloride levels and by serum. Thus, administration of TAP may not be effective for prevention of *M*. *haemolytica* pneumonia.

## Introduction

Bovine respiratory disease (BRD) is the most economically important disease affecting production in the beef industry. Methods of control include mass medication with antibiotics when cattle arrive at backgrounding or finishing operations, pre-conditioning and pre-vaccination programs, and early recognition and therapy of affected animals. Metaphylactic use of antibiotics is effective but it does not address the underlying roles of stress and viral infection. Moreover, mass antibiotic use can adversely impact consumer perception of beef production, and may lead to development of antibiotic-resistant animal and human pathogens. Pre-conditioning and pre-vaccination can also be effective but implementation is problematic because the costs may not be recovered by increased selling price. Thus, despite advances in preventive methods for this important disease, safe and cost-effective alternatives to control BRD are needed.

Risk factors for BRD are widely recognized and include viral infections and the stresses of weaning, transportation, castration and inclement weather conditions—all of which may occur at the time calves are removed from their farm-of-origin and enter feedlots. We have shown that well-recognized factors predisposing cattle to bacterial pneumonia, including stress (modeled by glucocorticoids) and viral infection (specifically bovine viral diarrhea virus, BVDV), interfere with the inducible expression of tracheal antimicrobial peptide (TAP), a β-defensin secreted by airway epithelial cells [1,2]. Together, these findings suggest that stress and viral infection may impair innate pulmonary defences by modulating the expression of epithelial antimicrobial peptides such as TAP.

We have shown, using two complementary methods, that the bacteria causing shipping fever pneumonia in cattle (*Mannheimia haemolytica*, *Histophilus somni*, and *Pasteurella multocida*) are susceptible to killing by TAP, and none of the bacterial isolates tested had developed resistance [3]. Moreover, the minimum inhibitory concentration (MIC, 1.56–6.25 μg/mL, 0.38–1.52 μM) was considered achievable in vivo in an inflammatory lesion. Although other immune defences may also be suppressed in stressed and virus-infected beef calves, the susceptibility of BRD pathogens to TAP suggests that restoring expression of this antimicrobial peptide may be an approach to preventing bacterial pneumonia.

Although TAP has been shown to have bactericidal effects in vitro, there are no reports of its activity in vivo. This study tested the hypothesis that in calves experimentally challenged with *M*. *haemolytica*, subsequent administration of TAP would reduce the number of surviving bacteria in the nasal cavity and lungs, and reduce the severity of the resulting pneumonia. The studies were conducted in calves because they are the target species of interest and are most relevant to this host-adapted pathogen. We used aerosol *M*. *haemolytica* challenge of clean-catch calves because the aerosol route mimics the natural infection and would not bypass the airway defences, and because this model does not require prior infection with viruses that could affect the respiratory defences. The study comprised three investigations. First, two forms of TAP were synthesized with or without oxidation of the disulfide bonds, and the bactericidal activities of these peptides were compared against the *M*. *haemolytica* strain used in the subsequent studies. Next, calves were challenged with *M*. *haemolytica* and the effect of synthetic TAP on bacterial infection and pneumonia was evaluated. Finally, to investigate possible explanations for the in vivo results, the effect of serum, sodium chloride and bronchoalveolar lavage fluid on in vitro antibacterial activity of TAP was studied.

## Materials and methods

### Synthetic tracheal antimicrobial peptide

Synthetic TAP was purchased (Biomatik, Cambridge, ON). Two peptides were prepared, with the same amino acid sequence “NPVSCVRNKG ICVPIRCPGN MKQIGTCVGR AVKCCRKK”, which corresponds to the peptide sequence previously named ‘20N’ [3]. The non-oxidized peptide had no disulfide bonds; the molecular weight was 4118.08 Da and the purity was 95%. The oxidized peptide had three disulfide bonds between Cys 5-Cys 34, Cys 12-Cys 27 and Cys 17-Cys 35; the molecular weight was 4112.08 Da and the purity was 80%. The peptides were supplied as dry trifluoroacetate salts and were reconstituted in double-distilled water.

### Isolation and propagation of *Mannheimia haemolytica*

*Mannheimia haemolytica* B158 was isolated from a cow with fatal pneumonia. The strain was identified as *M*. *haemolytica* genotype 2 by MALDI-TOF mass spectrometry (Animal Health Laboratory), as serotype 1 by PCR, and shown to carry the *M*. *haemolytica* leukotoxin gene as determined by qPCR [4]. Bacteria were grown to mid-log phase as previously described [4] and resuspended in 10 mM monobasic sodium phosphate buffer (pH 7.4). The concentration was estimated based on the optical density measured at 620 nm and diluted accordingly to obtain the desired concentration.

### Bactericidal activity of oxidized and non-oxidized TAP

To determine if the three disulphide bonds in oxidized TAP affected the antibacterial properties of the peptide, the bactericidal activity of synthetic oxidized and non-oxidized TAP was measured against *M*. *haemolytica* B158 [5]. Bacteria were prepared as described above, then 50 μL of bacteria at 2×10^4^ CFU/mL (i.e., 10^3^ CFU) were exposed to various concentrations of oxidized and non-oxidized TAP (2-fold dilutions from 100 to 1.56 μg/mL [24.28 to 0.38 μM], in monobasic sodium phosphate buffer, 100 μL final volume, in triplicate) for 1 h at 37°C, then plated onto blood agar and incubated at 37°C for 24 h. The number of colonies was counted in all samples at time 0, in controls without TAP, and samples with oxidized or non-oxidized TAP. The MIC was determined as the minimum final concentration of TAP that resulted in no bacterial growth on the blood agar plates.

### Clean-catch calves

All studies were approved by the University of Guelph Animal Care Committee (#3286) and conducted in accordance with the guidelines of the Canadian Council on Animal Care. Calves were raised by experienced and trained animal care staff, and handling and procedures were conducted by veterinarians with training and experience in the procedures (or veterinary students under direct veterinary supervision). Clean-catch calves free from *M*. *haemolytica* were obtained as previously described [4]. Briefly, calves were taken immediately after birth without any contact with the dam and raised in groups of 2 animals per room in isolation. They were treated with 2 mg/kg ceftiofur (Excenel, Zoetis, Kirkland, QC; subcutaneously) daily for the first 7 days of life and once with vitamin E and selenium (Dystosel, Zoetis, Kirkland, QC; 1 mL, subcutaneously). Each newborn calf was given a mixture of 1 L (i.e, half of the recommended dose) of the same batch of commercial colostrum (Instant Mix, SCCL, Saskatoon, SK) and 1 L of milk replacer (Grober High Conversion 20/15, Grober Nutrition, Cambridge, ON) as the first feeding. After 12 hours, the calves were fed milk replacer 3 times per day. Hay and non-medicated calf-starter pellets (Sharpe Farm Supplies, Guelph, Ontario) were provided ad libitum starting from the third week of age.

### Experimental infection studies

In all studies, pre-challenge levels of serum antibodies to *M*. *haemolytica* leukotoxin were measured by ELISA (Prairie Diagnostic Services, Saskatoon, SK) as previously described [6,7]. Plasma fibrinogen, serum haptoglobin and complete blood counts were measured prior to the challenge and daily thereafter (Animal Health Laboratory, University of Guelph); these are indicators of sepsis that change in response to the systemic effects of *M*. *haemolytica* infection in the lung [4]. Right and left nasal swabs were obtained before the challenge and daily thereafter, using a cotton swab on a wooden applicator. Each swab was inserted into the nasal cavity to approximately the level of the medial canthus of the eye, gently moved back and forth, then retracted. The swabs were evaluated by aerobic bacterial culture and the number of *M*. *haemolytica* bacteria were scored as 1+, 2+, 3+, or 4+ (Animal Health Laboratory, University of Guelph).

At 1 month of age, calves were challenged with *M*. *haemolytica* B158 and treated with either TAP or sterile distilled water. Briefly, bacteria (confirmed by MALDI-TOF and qPCR; third in vitro passage) were prepared from frozen stocks, propagated until an optical density (620 nm) of 0.5 was reached (i.e., at log phase of growth), centrifuged, and resuspended to the desired concentration in PBS based on the OD_620_ (Table 1). A Gram-stained smear of the inoculum was examined microscopically to confirm a pure population of gram-negative short rods. The *M*. *haemolytica* inoculum was delivered using an air pump, nebulizer and face mask [4]. In a prior study, delivery of ink using this apparatus was shown to deposit particles in the nasal cavity, bronchi, bronchioles and alveoli [4]. Synthetic non-oxidized TAP or water was delivered three times at 20 minutes, 2 hours and 6 hours after *M*. *haemolytica* challenge as described below. These time points were based on knowledge that TAP-induced killing of *M*. *haemolytica* occurs within 30–60 minutes in vitro (S9 Fig), but with the expectation that aerosol or intranasally delivered TAP would be cleared by the mucociliary apparatus.

**Table 1:** Overview of the design and bacteriology and pathology findings of the *in vivo* experiment.

**Calf pair**	**Prechallenge antibody level**^**1**^		**Bacterial challenge**^**2**^		**Euthanasia date (DPI)**^**3**^	**Nasal** *M*. *haemolytica*^**4**^ **(days positive/ days tested)**		**Lung** *M*. *haemolytica* ^**5**^ **(maximum CFU/100 mg)**		**Gross lung lesions**^**6**^ **(%)**		**Lung:heart weight ratio**	
	TAP	Water	OD	CFU		TAP	Water	TAP	Water	TAP	Water	TAP	Water
1	1300	300	1.0	5x10^10^	1	1/1	1/1	0	2.0x10^9^	1.1	10.0		
2	900	500	0.5	2x10^9^	3	2/3	3/3	4.5x10^6^	2.0x10^2^	7.7	0.1	2.00	1.92
3	1000	1600	0.5	4x10^9^	4	2/4	3/4	0	0	0.1	0.7	1.65	2.15
4	3200	3100	0.5	2x10^9^	2	2/2	2/2	0	2.0x10^8^	1.1	22.0	1.92	2.13
5	2100	2800	0.7^7^	9x10^8^	7	4/4	3/4	2.5x10^7^	5x10^4^	3.7	0.9	2.33	1.93
6	3900	5400	0.5	2x10^9^	4	4/4	4/4	0	0	0.7	0.1	1.82	2.23

^1^ Prechallenge antibody levels: the data suggest an absence of antibody to *M*. *haemolytica* leukotoxin, as the ELISA values were similar to those in fetal calf serum and lower than the range reported in conventional calves [4].

^2^ Bacterial challenge: quantification of *Mannheimia haemolytica* used for the challenge, based on the intended OD (600 nm) and by subsequent colony counts on agar (CFU administered to the calf).

^3^ Euthanasia date: calves were euthanized on the indicated number of days post-infection.

^4^ Nasal *Mannheimia*: nasal swabs were obtained dailyfor culture, and *Mannheimia haemolytica* was identified by colony morphology. The data show the number of days with positive cultures.

^5^ Lung *Mannheimia*: samples of cranioventral, caudoventral and caudodorsal lung were obtained at postmortem (plus sampling lesions when present), quantitatively cultured, and the number of *Mannheimia* colonies per 100 mg of lung tissue was determined.

^6^ Gross lung lesion score: The percentage of lung tissue affected by consolidation based on image analysis.

^7^ Calf pair #5 were challenged on day 0 (OD = 0.5, 9x10^8^ CFU), then re-challenged on day 3 with 1x10^10^ CFU, and euthanized on day 8 post-infection. Only the clinical data prior to the time of re-challenge are presented.

Calves were assessed without knowledge of treatment status at approximately 2, 4, 6, 10 and 12 hours after infection. After the first 12 hours calves were assessed four times per day. At these times, rectal temperatures and heart and respiratory rates were recorded and the following clinical signs were scored: demeanor (0–4), strength (0–4), respiratory effort (0–3), and cough (0–3), as well as appetite (0–3) at the most recent feeding (S1 Table). The clinical score (maximum 17) was the sum of these 5 individual scores.

Calves were immediately euthanized before the planned endpoint if any of the following scores were reached: demeanor 3, strength 3, respiratory distress 3, or anorexia at 2 consecutive feedings (S1 Table). All surviving animals (except pair 5, described below) were euthanized at 4 days after infection at the planned end point of the study. No animals died prior to euthanasia. This challenge protocol was intended to induce relatively mild clinical disease, with quantification of postmortem lung lesions as the major outcome of interest. Calves were euthanized at 4 dpi rather than later time points to permit quantitative lung culture and examination of lung lesions during the early disease period, because the study objectives focused on acute antimicrobial effects of TAP in the acute stages of infection.

At postmortem examination, the lungs (excluding the trachea) and heart (cut off at the heart base) were weighed separately, and the lung:heart weight ratio was calculated. The lung:heart weight ratio was intended as an objective estimate of the amount of edema and cellular exudate filling the airspaces of the lung; heart weight was used as an estimate of body size because body weight varies with the amount of rumen fill. The lungs were evaluated visually and by palpation without knowledge of the treatment status. Photographs of the dorsal and ventral surfaces of the lungs, and the laterodorsal surface of the right and left lungs were analyzed by image analysis (Image J software, National Institute of Health, Bethesda, MD) to determine the percentage of the right and left lung affected by pneumonia. Samples were harvested from right and left cranioventral, caudodorsal and caudoventral areas of lung as well as accessory lung lobe, obtaining samples from areas of pulmonary consolidation where possible. Samples were retained on ice for quantitative bacterial culture, and specimens from the same areas were placed in formalin for histopathology, in addition to tissues from any other areas with lesions.

Six pairs of calves were studied (Table 1). The first pair of calves received 5x10^10^ CFU of *M*. *haemolytica*. As this caused severe disease, a target dose of 2x10^9^ CFU was used for the later pairs. In the first two pairs of calves, 1 mg of TAP in 2 mL water was administered by aerosol 3 times (3 mg total). This dose was estimated to be 32 times the MIC of 6.25 μg/mL against *M*. *haemolytica* in vitro [3] and an estimated 20 mL maximal volume of epithelial lining fluid in the respiratory tract. The last 4 pairs of calves were given a larger TAP dose of 4 mg in 2 mL water by aerosol and 1 mg in 2 mL water by bilateral intranasal mist at each of the 3 treatments (15 mg total) using an atomizer device (MED-RX, Benlan Inc., Oakville, ON). Three pairs were euthanized before the planned endpoint because of severe disease: the first pair, at 1 day after infection; the second pair, at 3 days after infection; and the fourth pair, at 2 days after infection. The third and sixth pairs were euthanized at the planned endpoint of 4 days after infection. Because the fifth pair of calves developed only equivocal clinical signs after challenge, they were re-challenged with a higher dose on day 3 after the first challenge and euthanized on day 8 after the first challenge. Only the clinical data prior to the time of re-challenge are presented. For the fifth pair, the postmortem lung data are also presented, because histologic examination showed the grossly observed lesions to be consistent with an effect of the first challenge 8 days earlier (based on the presence of early fibroplasia with immature collagen) and not consistent with an effect of the second challenge 3 days earlier.

### Effects of sodium chloride, serum and bronchoalveolar lavage fluid on bactericidal activity of TAP

To investigate the reason for the in vivo findings, the effects of sodium chloride, bovine serum and bovine bronchoalveolar lavage fluid (BALF) on bactericidal activity of TAP against *M*. *haemolytica* B158 were tested in vitro. Non-oxidized TAP at two concentrations (6.5 μg/mL and 12.6 μg/mL; i.e. 0.5 x MIC and 1x MIC, as determined in the above studies) was prepared in sterile monobasic sodium phosphate buffer (pH 7.4). Bacteria were prepared as described above to a concentration of 2x10^4^ CFU/mL. To measure the effect of sodium chloride, 50 μL of the bacterial suspension in sodium phosphate buffer (10^3^ CFU, final concentration) was added to 2-fold dilutions of sodium chloride (25 μL, final concentrations ranged from 200 to 1.5 mM), and TAP in sodium phosphate buffer (25 μL, 6.5 μg/mL and 12.6 μg/mL, final concentrations) was immediately added in a final volume of 100 μL. For negative and positive controls, the same number of bacteria were mixed with 2-fold dilutions of sodium chloride in sodium phosphate buffer without TAP, or with the same concentrations of TAP in sodium phosphate buffer without sodium chloride. The mixtures were incubated for 1 hour at 37°C to allow TAP-induced bacterial killing; then inoculated onto blood agar plates and incubated at 37°C for 24 h. The numbers of CFU were counted, and compared to the number of CFU in samples prior to incubation and in control groups without TAP. All experiments were done twice in triplicate.

The effects of bovine serum and bovine BALF were assessed as follows. The sterility of fetal bovine serum was confirmed by plating 100 μL of the sample onto blood agar and incubating at 37°C for 24 h. Bronchoalveolar lavage fluid obtained from healthy calves at the Livestock Research and Innovation Centre Dairy Facility (LRICD, also known as Elora Dairy Research station) was centrifuged for 10 minutes at 500 x *g* to remove cells, then concentrated by centrifugal filtration with a 3-kDa molecular weight–limit device (Amicon Ultra-15, Millipore, Billerica, MA) and purified using a Ready Prep 2-D Cleanup Kit (Bio-Rad Laboratories Inc, Hercules, CA). The BALF was sterilized by filtration (0.2 μm) and sterility was confirmed by inoculating 100 μL onto a blood agar plate. The final protein concentrations of the serum and of the concentrated BAL fluid were measured (Bio-Rad protein Assay, Bio-Rad Laboratories, Hercules CA). *Mannheimia haemolytica* bacteria (10^3^ CFU in 50 μL, prepared as described above) were added to 2-fold dilutions (25 μL) of either sterile bovine serum (final concentrations from 2.5% to 0.0097%) or sterile bovine BALF (final concentrations from 100% to 0.78%), and the indicated concentrations of TAP (25 μL) were immediately added to reach the final volume of 100 μL. For positive and negative controls, the same number of bacteria were mixed with 2-fold dilutions of serum or BALF in monobasic sodium phosphate buffer without TAP, or with the same concentrations of TAP in monobasic sodium phosphate buffer without serum or BALF. The mixtures were analyzed as above.

### Statistical analysis

The in vitro experiments and the postmortem data from in vivo experiments were statistically analyzed using Prism 8 (GraphPad, San Diego, CA). Data were tested for normal distribution using the D'Agostino & Pearson normality test. If needed, data were log-transformed to achieve a normal distribution or tested using a non-parametric test. The data sets were analyzed by paired t test or by 2-way ANOVA depending on the number of parameters. P values less than 0.05 were considered statistically significant. Data in graphs are shown as mean ± standard error of the mean unless otherwise indicated.

Antemortem data from the in vivo experiments were statistically analyzed with SAS (version 9.4, 2013; SAS Institute Inc., Cary, NC) using calf (n = 12) as the experimental unit. Proc UNIVARIATE (SAS version 9.4) was used to assess the distribution of all data. Total leukocyte, monocyte, and segmented neutrophil counts were log-transformed to fit a normal distribution. All blood parameters were modeled separately as a general linear mixed model using Proc GLM (SAS version 9.4) and the individual fixed effects of pair, day relative to challenge, treatment, and treatment*day interactions were analyzed.

## Results

### Bactericidal activity of oxidized and non-oxidized TAP

**Fig 1:**
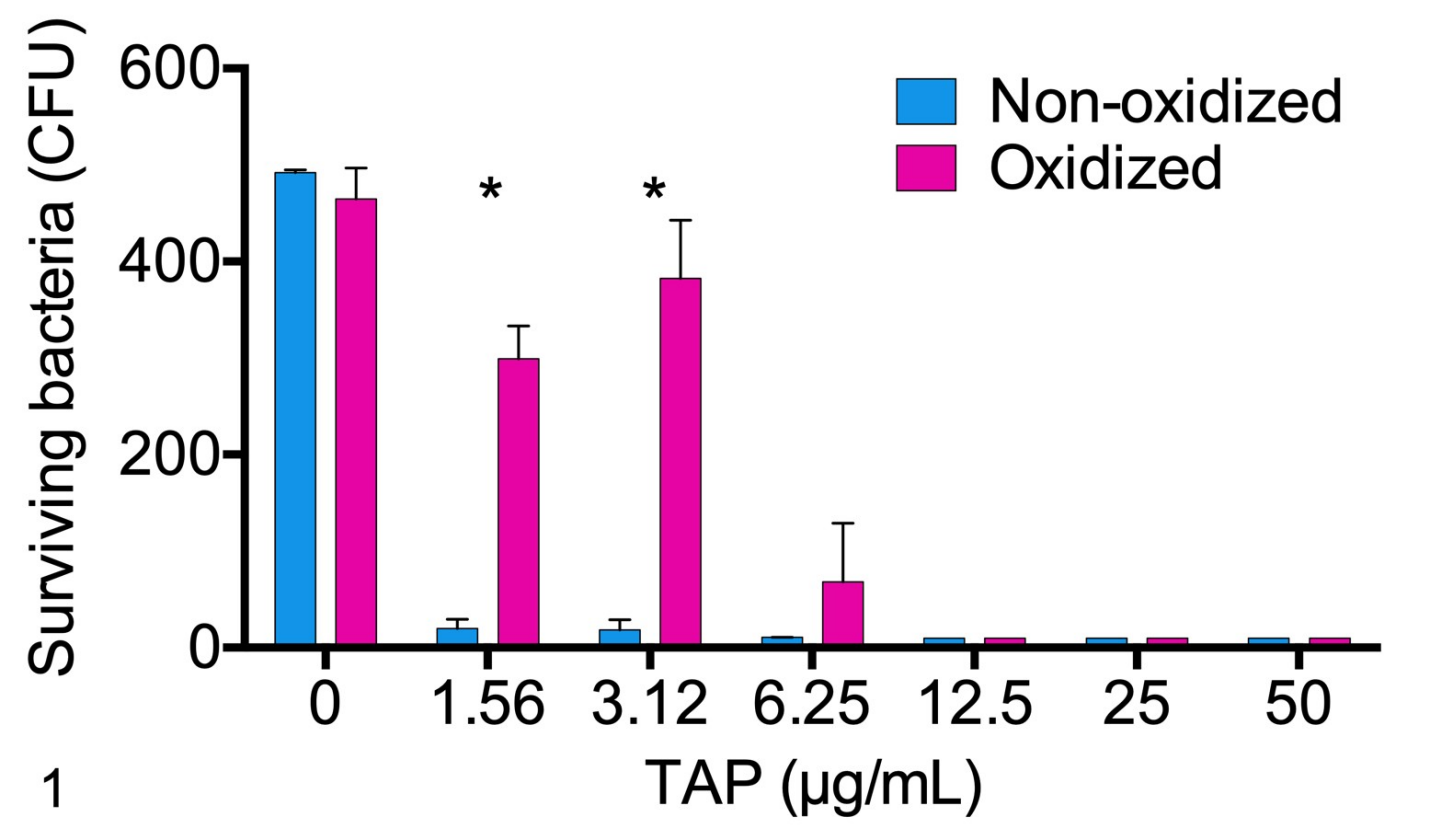
Comparison of the bactericidal activity of non-oxidized and oxidized tracheal antimicrobial peptide (TAP) against *Mannheimia haemolytica* B158. Serial dilutions of TAP in monobasic sodium phosphate buffer were incubated with *M*. *haemolytica* for 1 hour. The preparation was inoculated onto blood agar plates, and colonies were counted the next day. *P<0.05 (non-oxidized vs oxidized TAP), 2-way ANOVA, n = 3 replicates per group.

To determine whether oxidized or non-oxidized TAP (i.e., with or without disulfide bonds) should be used for the in vivo studies, the in vitro antibacterial activities of oxidized and non-oxidized TAP against *M*. *haemolytica* B158 were compared. The effects of TAP, oxidation status, and their interaction were all significant (P<0.001, 2-way ANOVA; Fig 1). Non-oxidized TAP had greater bactericidal activity than oxidized TAP at concentrations of 1.56 and 3.12 μg/mL. At concentrations of 3.12 and 6.25 μg/mL, non-oxidized TAP killed 96% and 99% of bacteria compared with 17% and 86% killing by oxidized TAP, respectively. At higher concentrations (12.5–50 μg/mL), both non-oxidized and oxidized TAP killed more than 99% of bacteria (P>0.05).

### Effect of TAP treatment on development of *M*. *haemolytica* pneumonia

**Fig 2:**
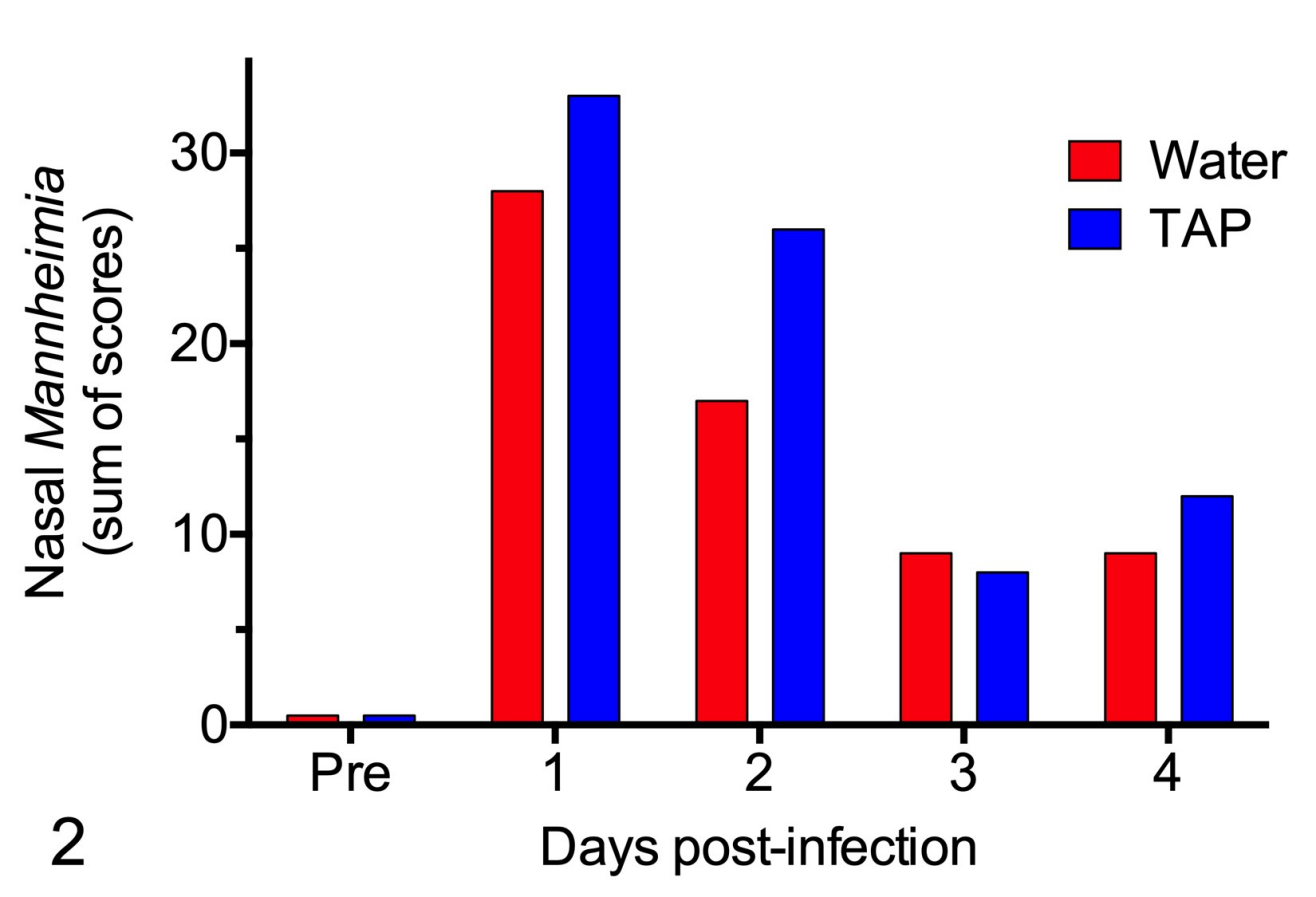
Isolation of *Mannheimia haemolytica* from right and left nasal cavities following challenge. Six pairs of calves were challenged with *M*. *haemolytica* B158 and treated with tracheal antimicrobial peptide (TAP) or water at 0.3, 2 and 6 hours after infection. Right and left nasal swabs were taken daily and the number of isolated *M*. *haemolytica* colonies was scored as 1, 2, 3 or 4 (maximum score of 8 for each calf). The data show the sums of scores across time for each group. The number of surviving calf pairs on each day are: n = 6 at 1 day post-infection, n = 5 at 2 days, n = 4 at 3 days, n = 3 at 4 days.

**Fig 3:**
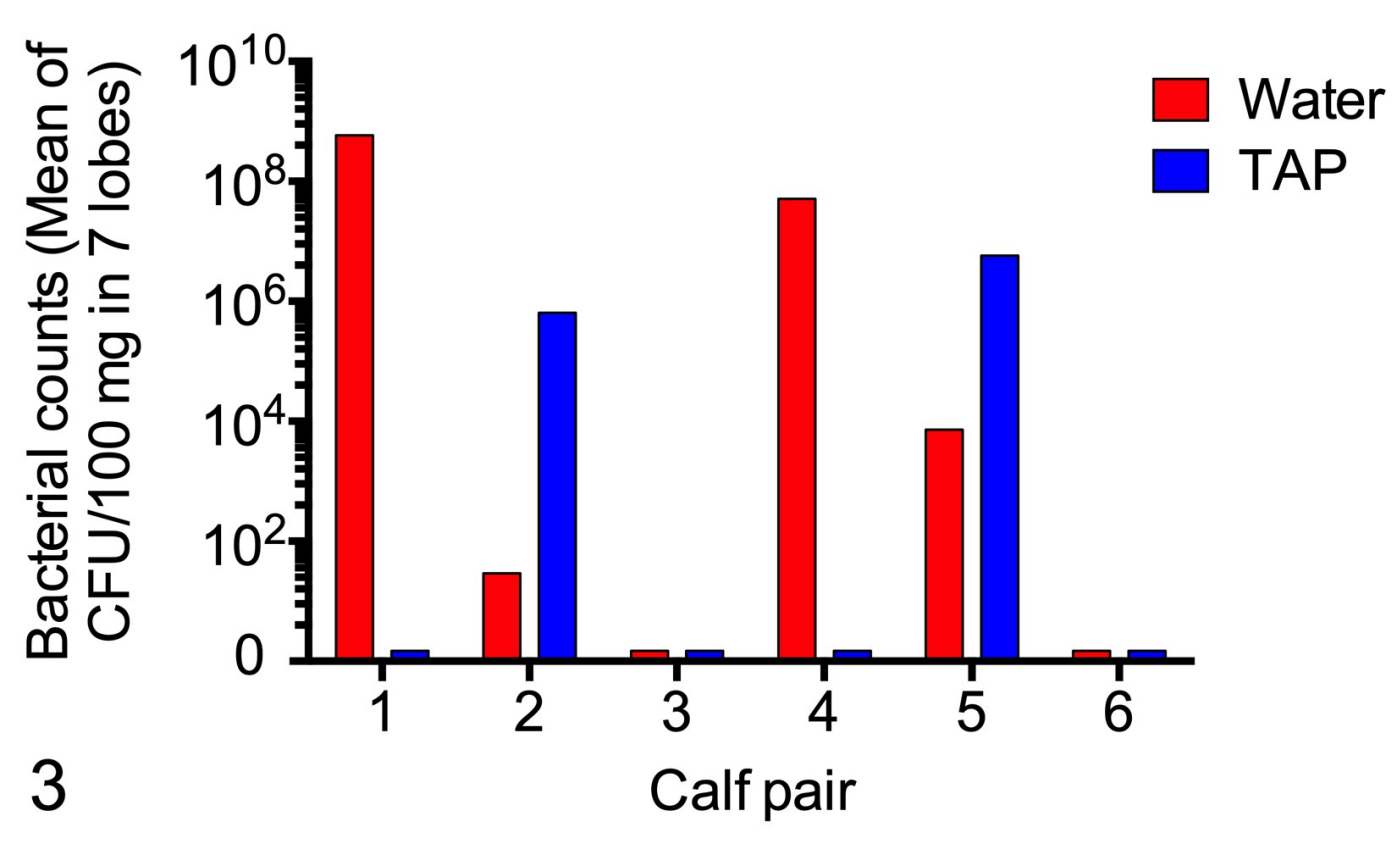
Isolation of Mannheimia haemolytica from the lungs. Six pairs of calves were challenged with M. haemolytica and treated with tracheal antimicrobial peptide (TAP) or water. At the end of the study, samples of right and left cranioventral, caudodorsal, and caudoventral areas of lung were analyzed by quantitative culture. The data show the mean bacterial counts among the 7 lobes tested for each calf.

Clean-catch calves (i.e. obtained prior to any contact with the dam), at least one month of age, were challenged by aerosol with *M*. *haemolytica* B158. *Mannheimia haemolytica* was isolated from the nasal cavity of all 12 challenged calves (Fig 2, S1 Fig). There were no consistent differences (*P =* 0.26) between water-treated versus TAP-treated calves with respect to isolation of *M*. *haemolytica* from the nasal cavity, when assessed as the number of time points with positive cultures or as the sum of the semiquantitative scores across the various time points (Fig 2, S1 Fig). At the time of postmortem examination, *M*. *haemolytica* was isolated from the lungs of 6 out of 12 challenged calves (Fig 3). The presence of *M*. *haemolytica* in the lung was not significantly different between water-treated and TAP-treated calves, when assessed as the lobe with the maximum number of bacteria isolated (*P =* 0.49, paired t test) or as the mean for all lobes (*P =* 0.49, paired t test, log-transformed data). The number of *M*. *haemolytica* per 100 mg of lung tissue was similar across the different lung lobes (S2 Fig).

**Fig 4:**
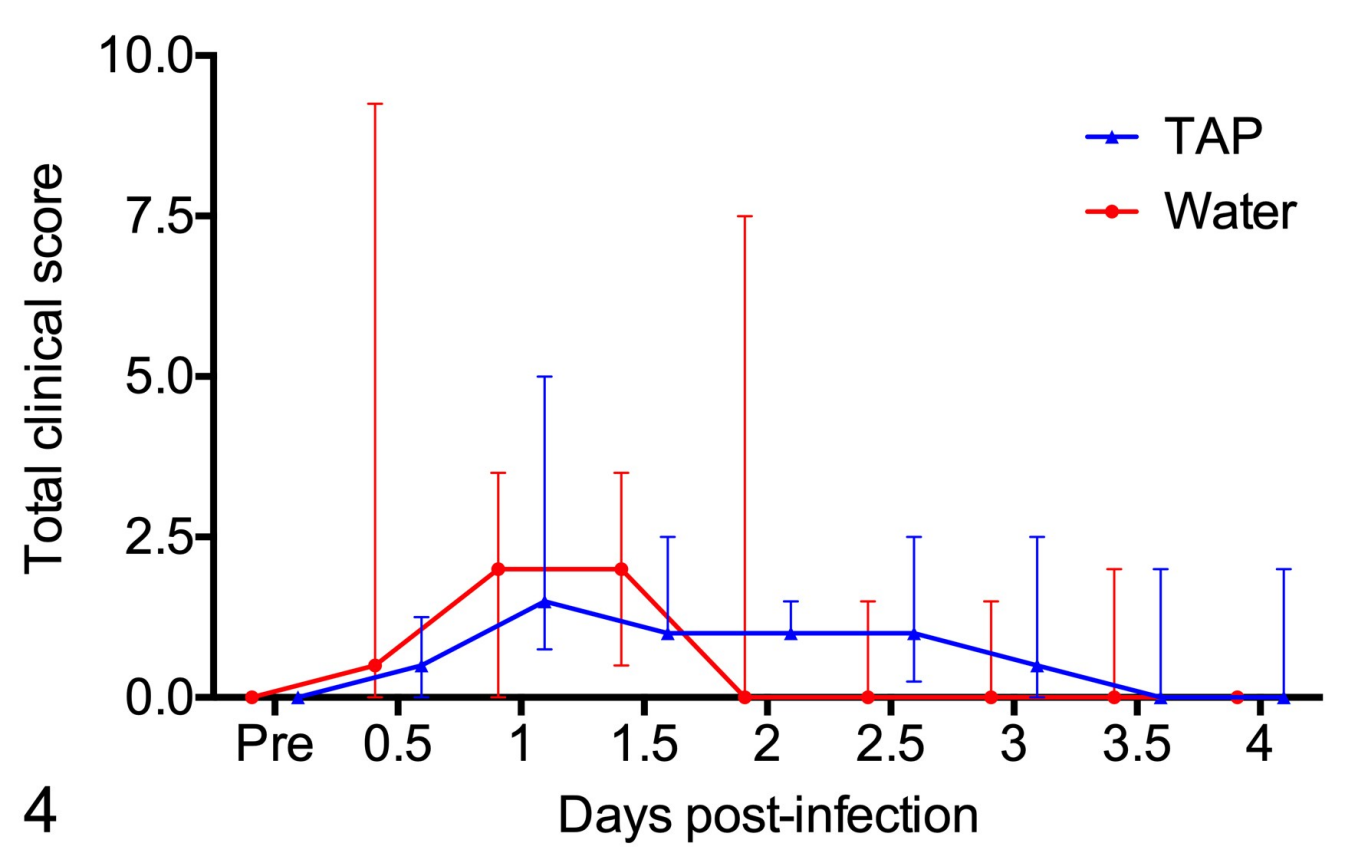
Clinical scores at different times after infection with *Mannheimia haemolytica*. Six pairs of calves were challenged with *M*. *haemolytica* and treated with tracheal antimicrobial peptide (TAP) or water. Clinical scores are the sum of individual scores for demeanor (0–4), strength (0–4), appetite (0–3), respiratory effort (0–3), and cough (0–3) for a maximum possible score of 17 at each time point. The data show the median and interquartile range for surviving calves. The number of surviving calf pairs on each day are: n = 6 at 1 day post-infection, n = 5 at 2 days, n = 4 at 3 days, n = 3 at 4 days. Since some pairs of calves were euthanized prior to the end point of 4 days post-infection, values may be compared between treatment groups but not among different time points.

Calves developed clinical signs following bacterial challenge including depression, increased respiratory rate, cough and inappetence, with recumbency and respiratory distress in the most severely affected calves. Increased clinical scores were observed within 12 hours post-infection in 6 of the 12 calves, and by 24 hours post-infection in another 5 calves; 1 calf did not show any clinical signs after challenge (Fig 4; S3 Fig). Of the six pairs of calves, three survived to the end of the study (calf pairs #3, #5, #6), two pairs were euthanized prior to the planned endpoint of the study because of clinical signs in the water-treated calves (calf pair #1, #4), and one pair was euthanized prior to the planned endpoint of the study because of clinical signs in the TAP-treated calf (calf pair #2). There were no consistent differences in clinical scores between calves that were treated with TAP or water after infection with *M*. *haemolytica* (Fig 4; *P =* 0.51).

**Fig 5:**
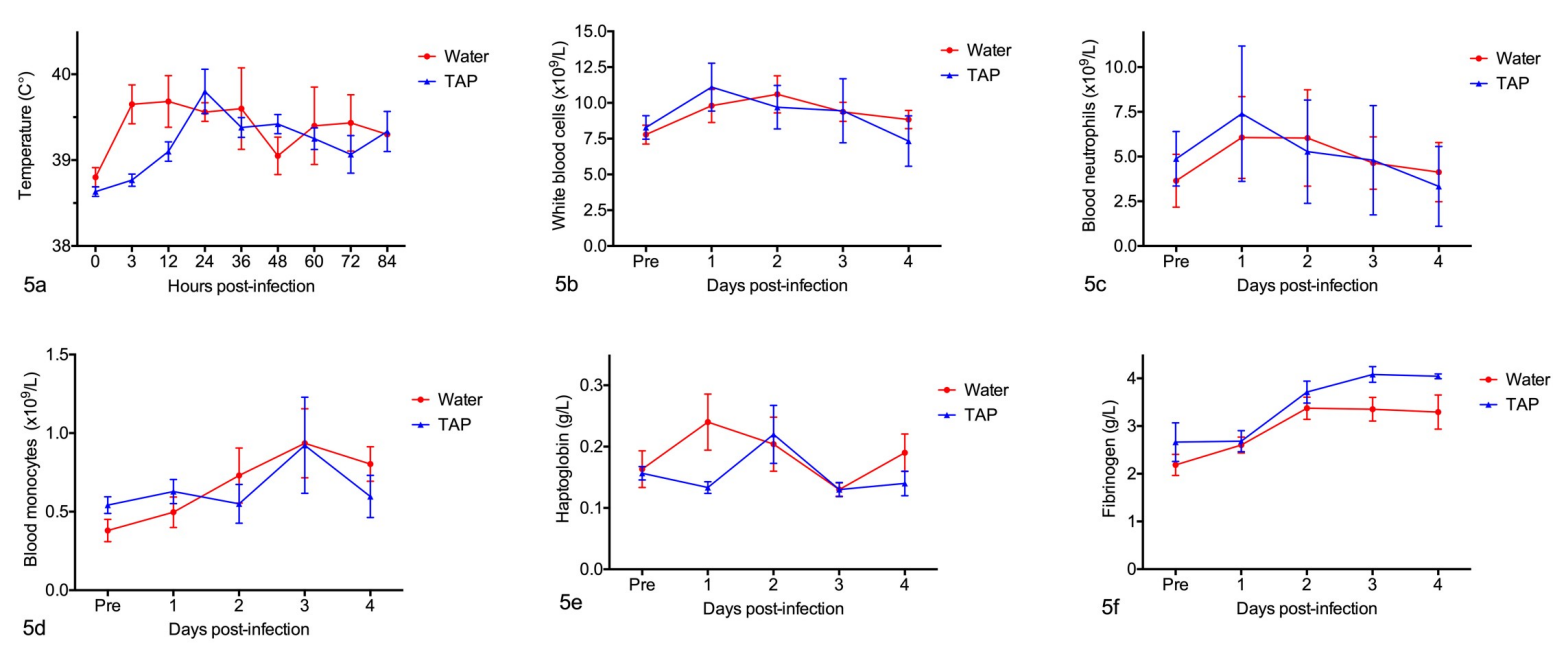
Rectal temperatures and clinical laboratory data at different times after infection with *Mannheimia haemolytica*. Six pairs of calves were challenged with *M*. *haemolytica* and treated with tracheal antimicrobial peptide (TAP) or water. Parameters were measured at the times shown before and after bacterial challenge. Mean ± standard error of the mean. (A) Rectal temperatures. (B) White blood cells. (C) Blood neutrophils. (D) Blood monocytes. (E) Serum haptoglobin. (F) Plasma fibrinogen. Since some pairs of calves were euthanized prior to the end point of 4 days post-infection, values may be compared between treatment groups but not among different time points.

Rectal temperatures prior to challenge ranged from 38.5–39.1°C. By 3 hours after infection, only 2 of 12 calves had a greater than 0.4°C increase in rectal temperature over pre-challenge values, but rectal temperatures increased in 8 of 12 calves by 12 hours after infection (Fig 5, S4A Fig). In general, infected calves had elevated numbers of white blood cells (*P =* 0.04), blood neutrophils (*P =* 0.002) and blood monocytes (*P =* 0.03), and increased concentrations of plasma fibrinogen (*P =* 0.0002) and serum haptoglobin (Fig 5; S4 Fig). There were no consistent differences between calves that were treated with TAP or water after infection with *M*. *haemolytica*, with respect to rectal temperatures (*P =* 0.29), white blood cell counts (*P =* 0.92), blood neutrophil (*P =* 0.75) or monocyte counts (*P =* 0.70), or serum haptoglobin concentrations. However, plasma fibrinogen concentrations tended to be higher in TAP-treated than water-treated calves (*P =* 0.03)(Fig 5).

**Fig 6:**
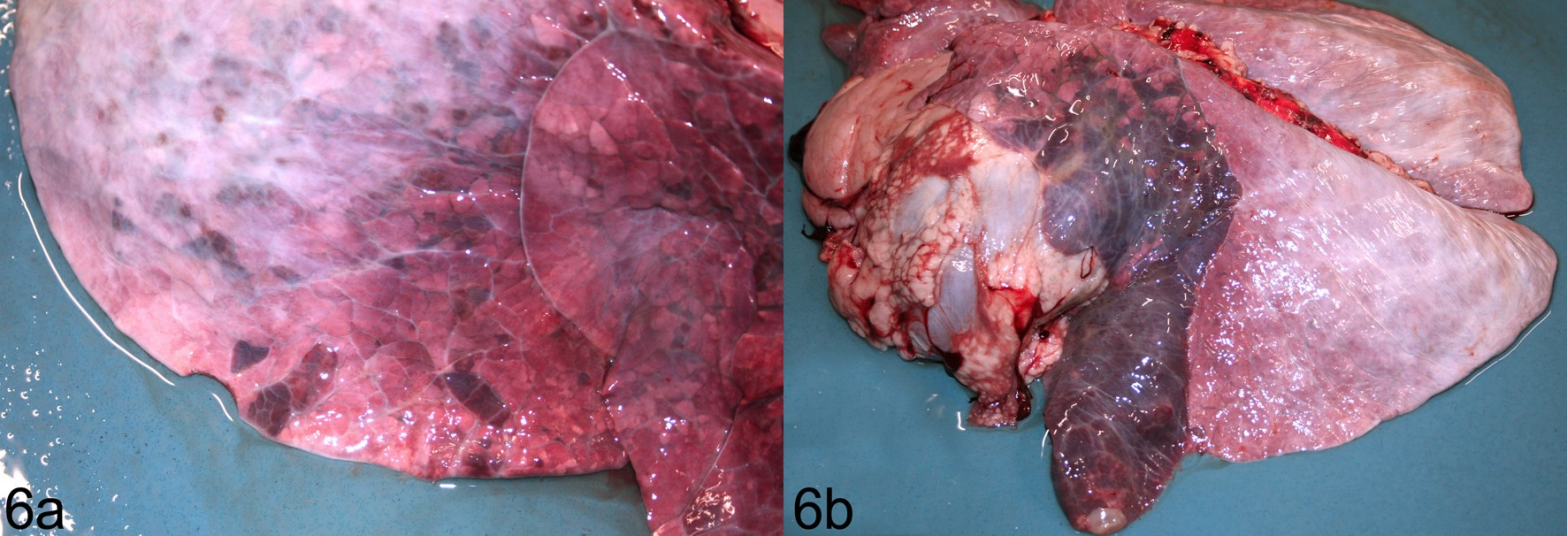
Gross lesions in calves challenged by aerosol with *Mannheimia haemolytica*. A) Lobular areas of red-purple color and firm texture. Lesions affect 15% of the right lung based on image analysis. Calf pair #1, water-treated (control). B) The left cranial lobe of the lung is almost entirely consolidated, red-purple, and collapsed. Lesions affect 39% of the left lung based on image analysis. Calf pair #4, water-treated.

**Fig 7:**
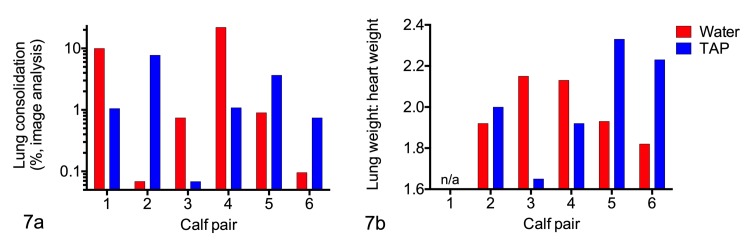
Quantitative analysis of gross lung lesions in calves challenged with *Mannheimia haemolytica*. Six pairs of calves were challenged with *M*. *haemolytica* and treated with tracheal antimicrobial peptide (TAP) or water. (A) Percentage of lung with lesions based on image analysis of lung photographs obtained postmortem. (B) Lung:heart weight ratios. n/a: not available (calf pair 1).

Gross lung lesions varied from tiny red foci to lesions that were angular, dark red-purple and firmer than normal lung (Fig 6A), to consolidation of an entire lung lobe (Fig 6B) or focal consolidation with fibrinous pleuritis (S5A Fig; S6 Fig). Gross lesions were most severe in the cranial lobes and right middle lobe, and mildest in the accessory lobe and the caudal lobes. In the TAP-treated calf that was challenged twice and euthanized at 8 days after the first infection, the lesions were multifocal nodules that resembled abscesses (S5B Fig). The TAP- and water-treated groups did not differ in the extent of gross lung lesions (Fig 7; image analysis, *P =* 0.94; lung:heart weight ratio: *P =* 0.84; paired t tests).

Histologically, the mildest lesions were neutrophil infiltration in bronchioles and alveoli with collapse of alveoli (S7 Fig). In more severe lesions, many of the leukocytes were necrotic and fibrin was present in alveoli (S7 Fig). The most severely affected sections of lung contained irregular areas of coagulation necrosis, surrounded by a densely basophilic rim of leukocytes that often had evidence of necrosis with streaming of the chromatin (i.e. “oat cells”) (S7 Fig). All 12 calves had histologic lesions of bronchopneumonia, and 7 of the 12 had oat cells and coagulation necrosis (i.e., lesions characteristic of *M*. *haemolytica* infection) in one or more histologic sections. There were no differences in these histologic parameters between TAP-treated and water-treated calves (S8 Fig).

Overall, the water-treated calves were more severely affected than the TAP-treated calves in 3 of the calf pairs (pairs 1, 3, 4) but less severe in the other 3 calf pairs (pairs 2, 5, 6).

### Effects of sodium chloride, serum and BALF on *in vitro* bactericidal activity of TAP

Tracheal antimicrobial peptide was mixed with various concentrations of sodium chloride, serum or BALF before incubation with *M*. *haemolytica* for 1 hour. In all of these experiments, 6.5 μg/mL TAP (without addition of sodium chloride, serum or BALF) killed 98–100% of the bacteria compared to the negative control (bacteria with buffer alone), while 12.5 μg/mL TAP killed 100% of the bacteria.

**Fig 8:**
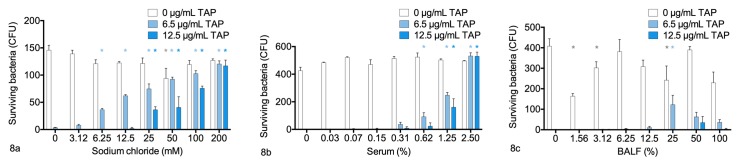
Effects of serum, sodium chloride, and bronchoalveolar lavage fluid (BALF) on survival of *Mannheimia haemolytica* B158 in the presence of tracheal antimicrobial peptide (TAP). Differing concentrations of (A) sodium chloride, (B) serum, or (C) concentrated BALF (diluted in monobasic sodium phosphate buffer) were mixed with 2 different concentrations of TAP or buffer before incubating with *M*. *haemolytica* for 1 hour. The mixtures were inoculated onto blood agar plates, and colonies were counted the next day. *P<0.05 compared to no sodium chloride, 2-way ANOVA, n = 3 replicates per group.

The addition of sodium chloride to TAP significantly increased the survival of *M*. *haemolytica* (P<0.0001, 2-way ANOVA, n = 3 technical replicates per group). This effect was observed with sodium chloride concentrations of 6.25 mM or greater in the presence of 6.5 μg/mL TAP, and with sodium chloride concentrations of 25.0 mM or greater in the presence of 12.5 μg/mL TAP. Sodium chloride concentrations of 100 mM and 200 mM resulted in 86% and 95% inhibition of the bactericidal activity of 6.5 μg/mL TAP (Fig 8A).

The addition of serum to TAP significantly increased the survival of *M*. *haemolytica* (P<0.0001, 2-way ANOVA, n = 3 technical replicates per group). This effect was observed with serum concentrations of 0.62% or greater in the presence of 6.5 μg/mL TAP, and with serum concentrations of 1.25% or greater in the presence of 12.5 μg/mL TAP. Addition of 1.25% serum resulted in 49% inhibition of the bactericidal activity of 6.5 mM TAP, and 2.5% serum increased bacterial numbers by 7% (Fig 8B). Lower concentrations of serum had no significant effect on the bactericidal activity of TAP.

The addition of concentrated BALF to TAP significantly increased the survival of *M*. *haemolytica* (*P =* 0.0009, 2-way ANOVA, n = 3 technical replicates per group). This effect was significant with 25% BALF in 6.5 μg/mL TAP (50% inhibition of bactericidal activity), but not with lower or higher BALF concentrations. BALF had no significant effect on the bactericidal activity of 12.5 μg/mL TAP (Fig 8C).

**Fig 9:**
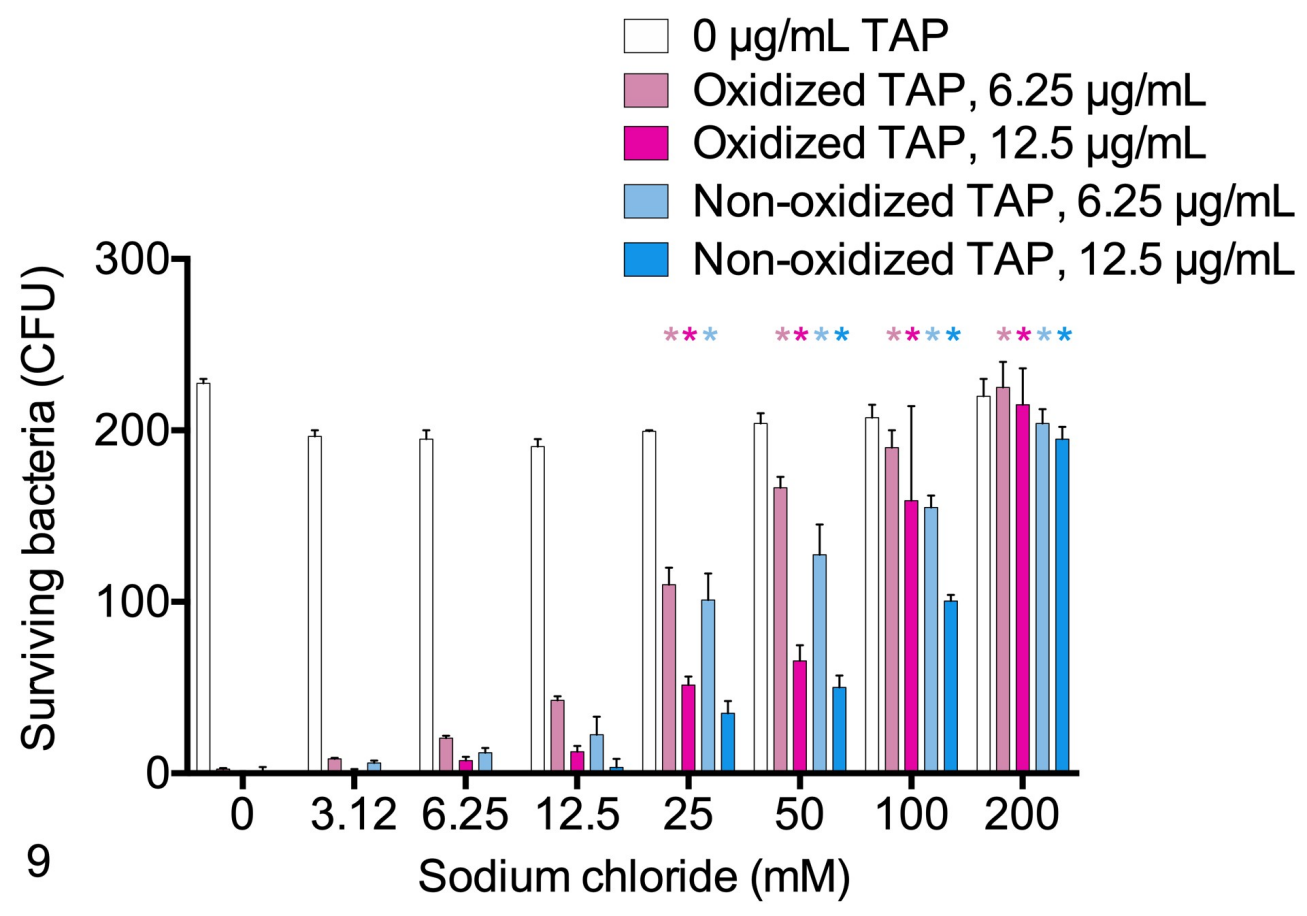
Effects of sodium chloride on survival of *Mannheimia haemolytica* B158 in the presence of oxidized or non-oxidized tracheal antimicrobial peptide (TAP). Different concentrations of sodium chloride were mixed with 2 different concentrations of oxidized or non-oxidized TAP or buffer before incubating with *M*. *haemolytica* for 1 hour. The mixtures were inoculated onto blood agar plates, and colonies (surviving bacteria) were counted the next day. *P<0.05 compared to no sodium chloride, 2-way ANOVA, n = 3 replicates per group. CFU, colony-forming units.

The effects of sodium chloride on the antimicrobial activities of oxidized and non-oxidized TAP were compared. Sodium chloride significantly inhibited the antimicrobial activity of both oxidized and non-oxidized TAP, reflected in increased survival of *M*. *haemolytica* (P<0.0001, 2-way ANOVA, n = 2 technical replicates per group). Using 6.5 mM TAP, 100 mM sodium chloride resulted in 92% and 75% inhibition of the bactericidal activity of oxidized and non-oxidized TAP, respectively. Overall, oxidized TAP had lower antimicrobial activity than non-oxidized TAP, in both the presence and the absence of sodium chloride (Fig 9). The number of remaining colonies of *M*. *haemolytica* were counted after being incubated for 0, 15, 30 or 60 minutes with either oxidized or non-oxidized TAP. The number of remaining colonies declined over time, and were greater for oxidized than non-oxidized TAP at all time points (S9 Fig).

## Discussion

Modulation of innate immune responses is a promising strategy for prevention of BRD [8]. There is increasing demand to develop alternatives to antibiotics for disease prevention in food production, in line with recent changes in policies for the use of medically important antimicrobials in Canada (eg, https://www.canada.ca/en/public-health/services/antibiotic-antimicrobial-resistance/animals.html) and elsewhere. Because TAP has bactericidal activity against the bacteria that cause BRD, we hypothesized that, after experimental challenge of calves with *M*. *haemolytica*, administration of exogenous TAP would reduce the number of surviving bacteria in the nasal cavity and lungs and reduce the severity of the resulting pneumonia. However, the results did not support this hypothesis, and we were further able to demonstrate that the bactericidal activity of TAP was inhibited by physiologic levels of sodium chloride and by the presence of serum in inflamed fluids lining the respiratory tract. These findings call into question the relevance of the in vitro bactericidal activity of TAP for controlling bacterial infections in vivo. Furthermore, the findings suggest that administration of exogenous TAP or stimulation of endogenous TAP expression in calves at risk of disease may not be an effective strategy for prevention of BRD, at least under the conditions used in these studies.

A comparison of the in vitro bactericidal activity of oxidized and non-oxidized TAP showed clear bactericidal activity that was dose-dependent. Non-oxidized TAP had significantly greater bactericidal effect against *M*. *haemolytica* B158 and was thus used used in the subsequent studies.

The use of clean-catch calves that received the same commercial colostrum resolved three main problems of researchers working with cattle as an in vivo model: the high frequency of *M*. *haemolytica* infection in conventional calves, the high prevalence of antibodies to *M*. *haemolytica* in conventionally raised calves, and the high susceptibility of colostrum-deprived calves to opportunistic infection. Compared to the previously reported model of aerosol *M*. *haemolytica* in clean-catch colostrum-deprived calves [4], these calves had a null or low antibody titer to *M*. *haemolytica* and a low occurrence of pre-challenge spontaneous infections. The calves in this study developed clinical, microbiologic and pathologic evidence of pneumonia following challenge with *M*. *haemolytica*, with similar clinical signs and gross and histologic lung lesions to the natural disease.

In contrast to the bactericidal activity of TAP identified in the in vitro studies, the in vivo studies did not show any significant bactericidal effect of TAP against *M*. *haemolytica*. Specifically, in nasal swabs obtained daily after challenge, the treatment groups did not differ in either the frequency with which *M*. *haemolytica* was isolated or in the semi-quantitative estimates of the number of CFU. Similarly, the treatment groups did not differ in the number of *M*. *haemolytica* colonies isolated from standardized samples of lung tissue at the end of the study.

Furthermore, administration of TAP did not protect against the development of pneumonia in the challenged calves. Among the 6 studied pairs of calves, in 3 pairs the TAP-treated animals showed milder clinical signs, lower clinical scores, and milder lung lesions, whereas in the other 3 groups of calves, disease was more severe in the TAP-treated animals.

The concentration of TAP delivered to the respiratory tract by aerosol was estimated to be 32 times the in vitro MIC for the first two pairs of calves, and 128 times the MIC for the last four pairs of calves (in addition to the intranasal mist of TAP delivered to the latter groups). Furthermore, calves receiving the higher TAP doses (calf pairs #3–6, 15 mg TAP) were not protected against disease. Thus, there was no evidence that underdosing of TAP contributed to the lack of a protective effect.

We considered that TAP might have differing roles in mild vs severe forms of pneumonia. In this study, the pairs of calves infected with a higher concentration of bacteria (calf pairs #1 and #3, based on CFU counts on the day following challenge) showed milder disease in the TAP-treated compared to the water-treated calf. Although we would not rule out a protective effect of TAP against high-dose infection, the number of animals in our study is not high enough to reliably prove this possibility.

The effects of some substances in the epithelial lining fluid of the respiratory tract (serum, sodium chloride and concentrated BALF) on in vitro TAP antibacterial activity were analyzed in an attempt to explain the lack of TAP bactericidal activity in vivo. Concentrated BALF had only minor effects on the bactericidal activity of TAP against *M*. *haemolytica*. Although lung epithelial lining fluid is more concentrated than our preparation of concentrated BALF, the findings suggest that BALF has minimal or no inhibitory effect on TAP function.

Bovine serum inhibited TAP bactericidal activity at concentrations of 0.62% and higher. We considered whether these findings might represent an effect of serum in promoting growth of *M*. *haemolytica* even in the face of bacterial killing by TAP. However, increasing amounts of serum in the absence of TAP did not increase the number of resulting CFU. Thus, the findings suggest that proteins or other constituents of serum inhibit the bactericidal activity of TAP, although only at relatively high concentrations.

In contrast to BALF and serum, sodium chloride completely inhibited TAP bactericidal activity in a dose-dependent manner with effects detected with sodium chloride concentrations as low as 6.25 mM. These findings suggest that physiological sodium chloride levels (154 mM) would reduce TAP bactericidal activity by an estimated 85–90% in the presence of 6.5 μg/mL TAP and by 63–77% with 12.5 μg/mL TAP. This is a novel finding for TAP. However, prior publications have reported in vitro salt sensitivity of other defensins [9,10]. Proposed mechanisms for the salt-sensitivity of defensins include interference with the electrostatic attraction of the cationic peptide to negatively charged bacterial components, or altered conformation or oligomerization of defensin peptides [11–13].

Based on these in vitro findings, the most likely explanation for the lack of in vivo protection by TAP is inhibition of its bactericidal activity by physiological levels of sodium chloride. Exudation of serum onto mucosal surfaces in the early stages of inflammation may also contribute to inhibition of TAP activity. Some previous reports support this suggestion. For example, alpha-helical antimicrobial peptides did not have an in vivo antimicrobial effect in murine models of *P*. *aeruginosa* infection, even though they were effective in vitro, suggesting that the pulmonary environment is not conducive to the function of the peptides [14]. In other research, human β-defensins demonstrated no in vivo antimicrobial activity, while LL-37 (a cathelicidin antimicrobial peptide), showed bactericidal activity in a murine model in synergy with tobramycin against *Staphylococcus aureus* and *Streptococcus* spp. [15]. On the other hand, recombinant human β-defensins killed *Salmonella* in vitro and protected against *Salmonella* infection in vivo when they were administered to mice [16]. Nonetheless, these studies overall support the interpretation that high sodium chloride concentrations contributed to the lack of a protective effect of non-oxidized TAP in our studies.

TAP is produced naturally by bovine airway epithelial cells, and is therefore presumed to have a beneficial in vivo function. Even though the bactericidal function of non-oxidized TAP was shown to be sensitive to physiologic sodium chloride concentrations, we measured whether the oxidized form (which may be more abundant in vivo) might be resistant to inhibition by salt. However, sodium chloride inhibited the bactericidal activity of both oxidized and non-oxidized TAP in vitro. These data do not completely rule out that oxidized TAP might have a bactericidal effect within the animal; however, since non-oxidized TAP had no measurable in vivo bactericidal effect against *M*. *haemolytica* (in nasal cavity or lung) and the in vitro bactericidal effect of oxidized TAP was inhibited by physiologic sodium chloride concentrations, we did not investigate this possibility with additional calf studies using oxidized TAP. Furthermore, it remains possible that oxidized TAP has some beneficial in vivo salt-resistant function (other than a bactericidal effect), even though non-oxidized TAP did not protect against disease under the conditions used in the present study.

The current study had some limitations typical of in vivo studies using calves. First, a restricted number of animals was used. However, the number of calf pairs with more severe disease in TAP-treated vs water-treated calves was similar to those with the converse outcome. This implies that even if there were a small protective effect, large numbers of experimental animals would be required to detect it, and such a small effect would not be an appropriate alternative to metaphylactic use of antibiotics to prevent BRD. Variability among calves could be considered as another limitation of the study. Despite following the same protocol in raising the calves, individual differences were nonetheless evident, although it should be noted that this variability of responses in genetically diverse research calves has been documented in other studies (4, 10). Second, although we did not identify a protective effect of TAP, we considered if this might depend on the severity of bacterial challenge. Specifically, in the 2 pairs of calves infected with a higher concentration of *M*. *haemolytica* (calf pairs #1 and #3), the TAP-treated calf developed milder disease compared to the water-treated calf. This remains an area for future investigation. Third, the apparatus used in this study delivered nebulized material to the nasal cavity, bronchi, bronchioles and alveoli, including ink in a prior study [4] and *Mannheimia haemolytica* in the present study. However, we were not able to quantify the concentration of TAP in the epithelial lining fluid of the lung, and this is a possible reason for the lack of therapeutic effect. Nonetheless, high levels of nebulized material did reach the nasal cavity even though the numbers of *Mannheimia haemolytica* in nasal swabs were similar in TAP-treated and water-treated calves. This suggests that failure to clear *Mannheimia*—at least from the nasal cavity—was due to inactivation of TAP rather than failure to achieve an adequately high TAP concentration.

In a *M*. *haemolytica* challenge model using clean-catch calves, treatment with non-oxidized TAP did not reduce bacterial loads in the respiratory tract or protect against disease. Sodium chloride and serum inhibited the in vitro bactericidal activity of TAP. These findings suggest that TAP does not control bacterial infections in vivo at mucosal surfaces because of interference by physiological sodium chloride levels and serum in inflamed tissues. Thus, administration of TAP to calves at risk of disease may not be effective for disease prevention in vivo.

## Supporting information

S1 Fig Isolation of *Mannheimia haemolytica* from right and left nasal cavities following challenge. Six pairs of calves were challenged with *M*. *haemolytica* B158 and treated with tracheal antimicrobial peptide (TAP) or water at 0.3, 2 and 6 hours after infection. Right and left nasal swabs were taken daily and the number of isolated *M*. *haemolytica* colonies was scored as 1, 2, 3 or 4. The data show (A) the number of days with positive *M*. *haemolytica* cultures, and (B) the sums of scores (across each time point) for each calf. Calf pairs were euthanized as follows: pair 1 at d1, pair 2 at d3, pair 3 at d3, pair 4 at d2, pair 5 at d8 (only data prior up to d4 are presented), pair 6 at d4.
(TIFF)

S2 Fig Isolation of *Mannheimia haemolytica* from lungs, by lobe. Six pairs of calves were challenged with *M*. *haemolytica* and treated with tracheal antimicrobial peptide (TAP) or water. At the end of the study, samples of right and left cranioventral, caudodorsal, and caudoventral areas of lung were analyzed by quantitative culture. The data show the number of CFU per 100 g of lung tissue in different lung lobes of individual calves.
(TIFF)

S3 Fig Clinical scores at different times after infection with *Mannheimia haemolytica*. Six pairs of calves were challenged with *M*. *haemolytica* and treated with tracheal antimicrobial peptide (TAP) or water. Clinical scores were determined as the sum of individual scores for demeanor (0–4), strength (0–4), appetite (0–3), respiratory effort (0–3), and cough (0–3) for a maximum possible score of 17 at each time point. The graph shows data for individual calves over time.
(TIFF)

S4 Fig Rectal temperatures and clinical laboratory data at different times after infection with *M. haemolytica*. Six pairs of calves were challenged with *M*. *haemolytica* and treated with tracheal antimicrobial peptide (TAP) or water. Parameters were measured at the times shown before and after bacterial challenge. Individual-animal data are shown. (A) Rectal temperatures. (B) White blood cells. (C) Blood neutrophils. (D) Blood monocytes. (E) Serum haptoglobin. (F) Plasma fibrinogen.
(TIFF)

S5 Fig Gross lung lesions in calves challenged by aerosol with *M. haemolytica*. (A) The right cranial lobe of the lung contains a focal area of consolidation with fibrinous pleuritis (arrow). Lesions affect 15.4% of the right lung based on image analysis. Calf pair #2, TAP-treated calf. (B) All lobes of the right lung contain round raised well-demarcated lesions that are developing into abscesses. Lesions affect 4.5% of the right lung based on image analysis. Calf pair #5, TAP-treated calf, 8 days after infection.
(TIF)

S6 Fig Gross lung lesions in all calves of the study. The images show the left and right lungs for the water-treated calf and the TAP-treated calf, for each calf pair in the study. Animal identification was blacked out for analysis. Image was missing for the left lung of water-treated calf #6 and analysis was based on the data collected at postmortem examination.
(TIF)

S7 Fig Histologic lesions in the lung of calves challenged by aerosol with *Mannheimia haemolytica*. Calf pair #4, water-treated, 3 days after infection.
(A) Well-preserved neutrophils fill a bronchiole (Br) and alveoli (Al). The alveoli are collapsed.
(B) Alveoli are filled with fibrin (F) and necrotic neutrophils (N).
(C) An inflamed lobule of lung contains a focal area of coagulation necrosis (CN) surrounded by a densely basophilic rim of leukocytes.
(D) The densely basophilic rim shown in image C contains necrotic leukocytes that have streaming of their chromatin (*) typical of oat cells.
(TIF)

S8 Fig Semi-quantitative scoring of histologic lung lesions in calves challenged with *M. haemolytica*. Grossly visible lesions were selectively sampled in three regions of lung: cranioventral, caudodorsal and caudoventral. Histologic lesions were semi-quantitatively scored based on bronchopneumonia (0–3), oat cells (0 or 2), and foci of coagulation necrosis (0–2). (A) The data show the sum of bronchopneumonia scores across the 3 sampled areas of lung. (B) The number of lung regions with oat cells (necrotic leukocytes with streaming chromatin). (C) The sum of coagulation necrosis scores across the 3 sampled areas of lung.
(TIFF)

S9 Fig Bactericidal activity of oxidized and non-oxidized tracheal antimicrobial peptide (TAP) over time. Different concentrations of oxidized or non-oxidized TAP or buffer were incubated with *M*. *haemolytica*. At 0, 15, 30 and 60 minutes, samples were inoculated onto blood agar plates, and the number of colonies (surviving bacteria) were counted the next day. CFU, colony-forming units.
(TIFF)

S1 Table Clinical scoring scheme and humane endpoints. The clinical score was the sum of these components. The maximal clinical score was 17.
(DOCX)

S2 Table Tables of raw data from the study. (XLSX)
