# Supplementary figures and images for: Effect of tracheal antimicrobial peptide on the development of Mannheimia haemolytica pneumonia in cattle
Source: PLoS One. 2019 Nov 26;14(11):e0225533. doi: 10.1371/journal.pone.0225533 (PMC6879128; doi:10.1371/journal.pone.0225533)

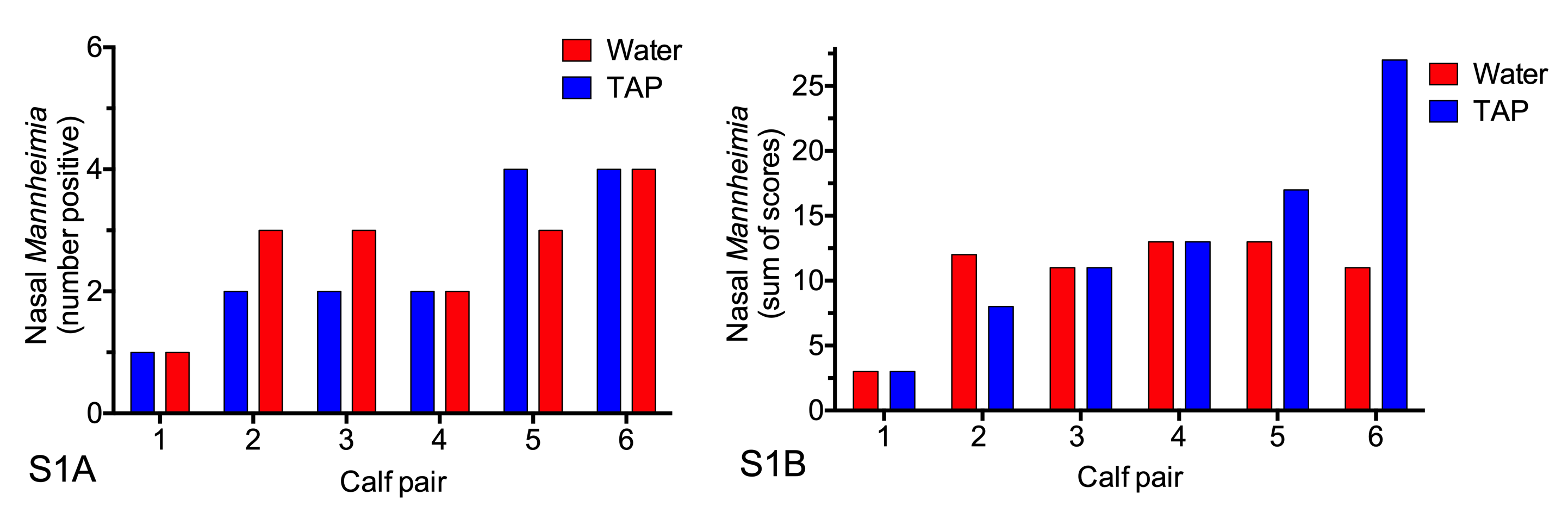

Supplement: S1 Fig — Six pairs of calves were challenged with M. haemolytica B158 and treated with tracheal antimicrobial peptide (TAP) or water at 0.3, 2 and 6 hours after infection. Right and left nasal swabs were taken daily and the number of isolated M. haemolytica colonies was scored as 1, 2, 3 or 4. The data show (A) the number of days with positive M. haemolytica cultures, and (B) the sums of scores (across each time point) for each calf. Calf pairs were euthanized as follows: pair 1 at d1, pair 2 at d3, pair 3 at d3, pair 4 at d2, pair 5 at d8 (only data prior up to d4 are presented), pair 6 at d4. (TIFF) [file pone.0225533.s001.tiff]

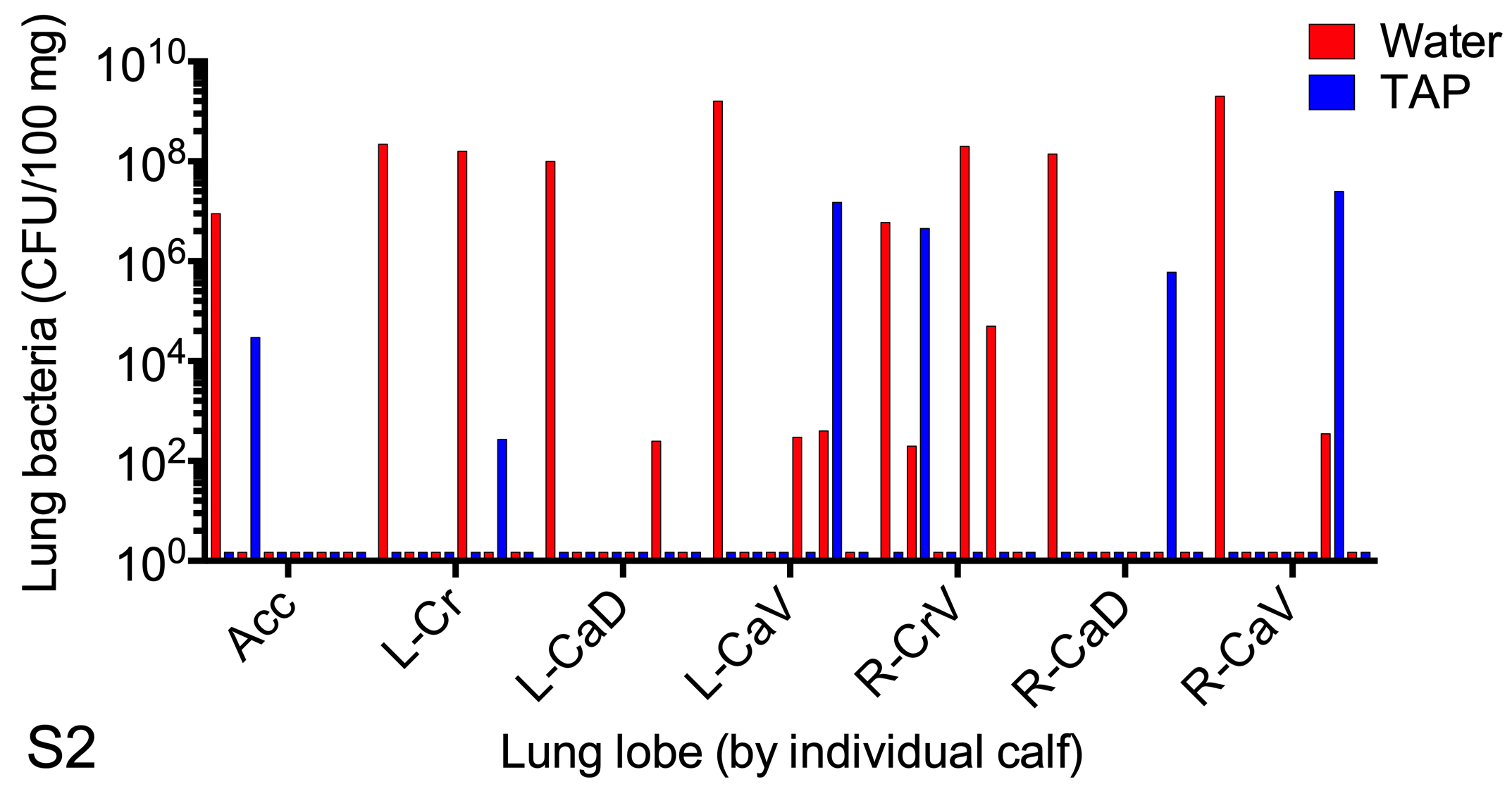

Supplement: S2 Fig — Six pairs of calves were challenged with M. haemolytica and treated with tracheal antimicrobial peptide (TAP) or water. At the end of the study, samples of right and left cranioventral, caudodorsal, and caudoventral areas of lung were analyzed by quantitative culture. The data show the number of CFU per 100 g of lung tissue in different lung lobes of individual calves. (TIFF) [file pone.0225533.s002.tiff]

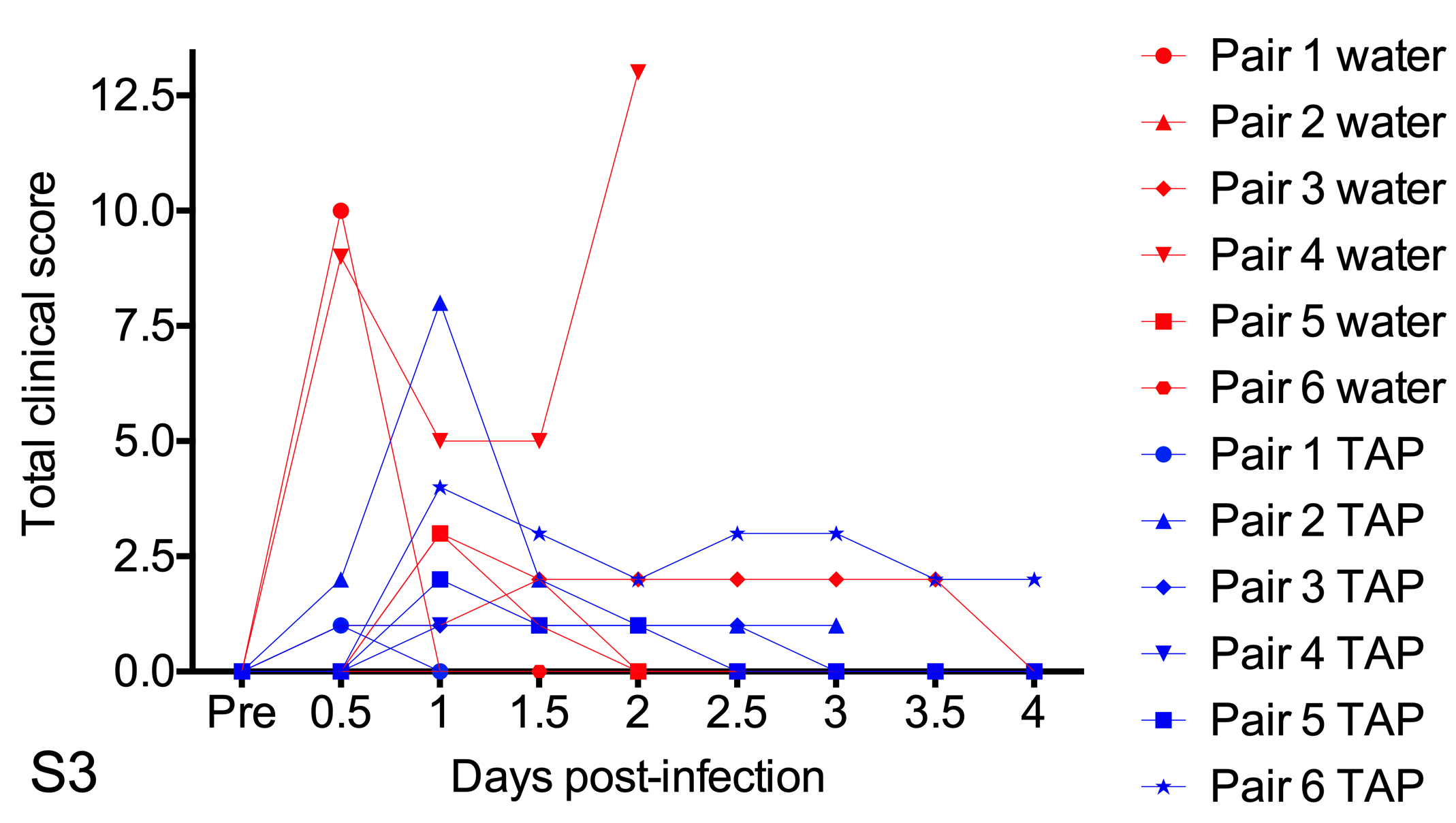

Supplement: S3 Fig — Six pairs of calves were challenged with M. haemolytica and treated with tracheal antimicrobial peptide (TAP) or water. Clinical scores were determined as the sum of individual scores for demeanor (0–4), strength (0–4), appetite (0–3), respiratory effort (0–3), and cough (0–3) for a maximum possible score of 17 at each time point. The graph shows data for individual calves over time. (TIFF) [file pone.0225533.s003.tiff]

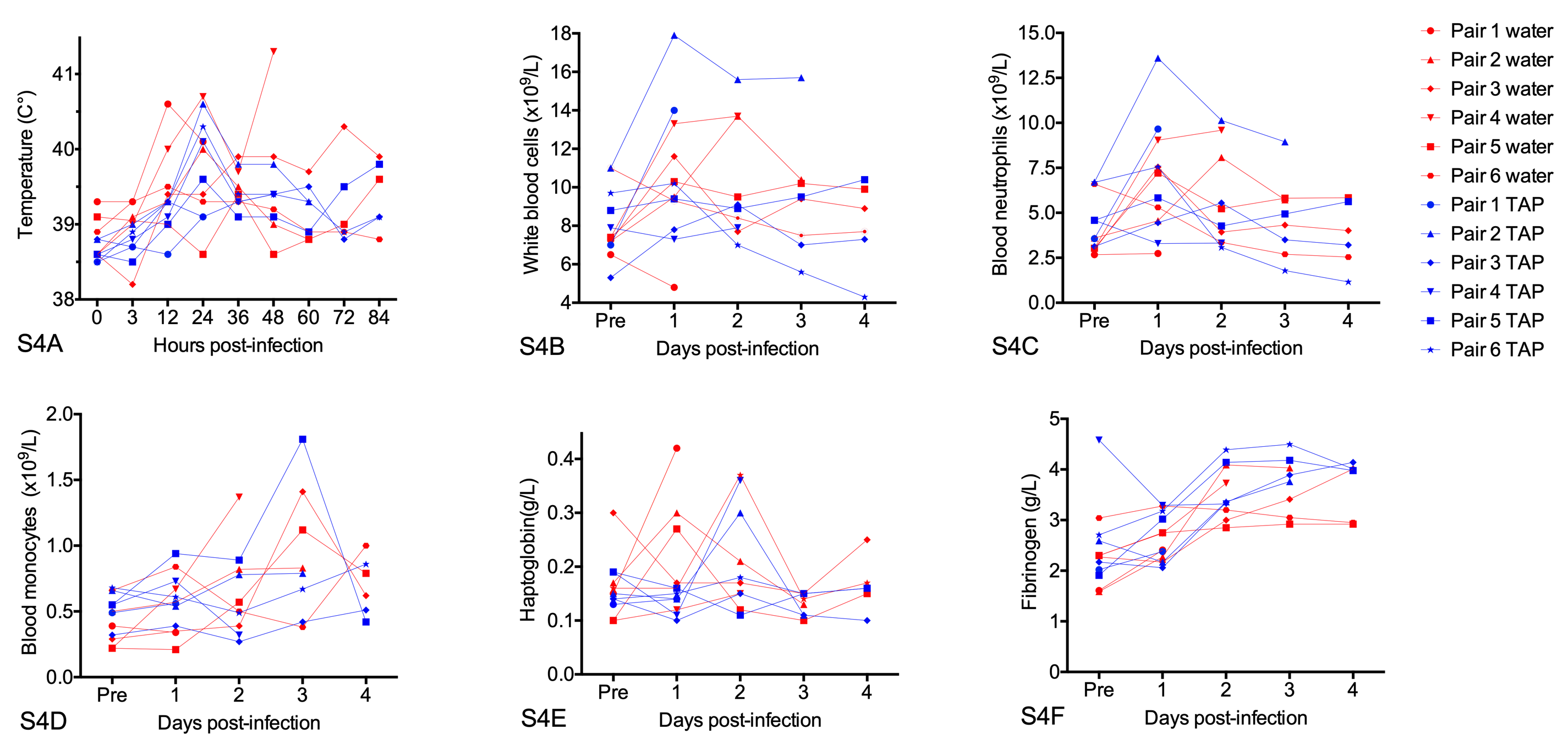

Supplement: S4 Fig — Six pairs of calves were challenged with M. haemolytica and treated with tracheal antimicrobial peptide (TAP) or water. Parameters were measured at the times shown before and after bacterial challenge. Individual-animal data are shown. (A) Rectal temperatures. (B) White blood cells. (C) Blood neutrophils. (D) Blood monocytes. (E) Serum haptoglobin. (F) Plasma fibrinogen. (TIFF) [file pone.0225533.s004.tiff]

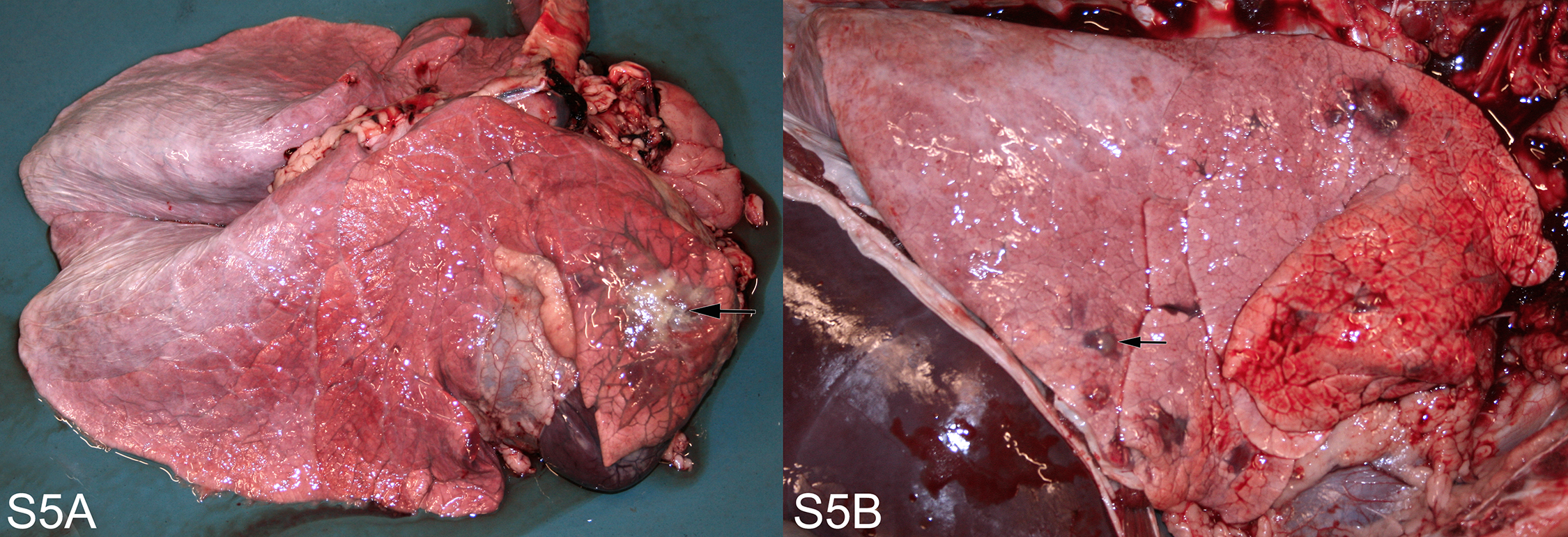

Supplement: S5 Fig — (A) The right cranial lobe of the lung contains a focal area of consolidation with fibrinous pleuritis (arrow). Lesions affect 15.4% of the right lung based on image analysis. Calf pair #2, TAP-treated calf. (B) All lobes of the right lung contain round raised well-demarcated lesions that are developing into abscesses. Lesions affect 4.5% of the right lung based on image analysis. Calf pair #5, TAP-treated calf, 8 days after infection. (TIF) [file pone.0225533.s005.tif]

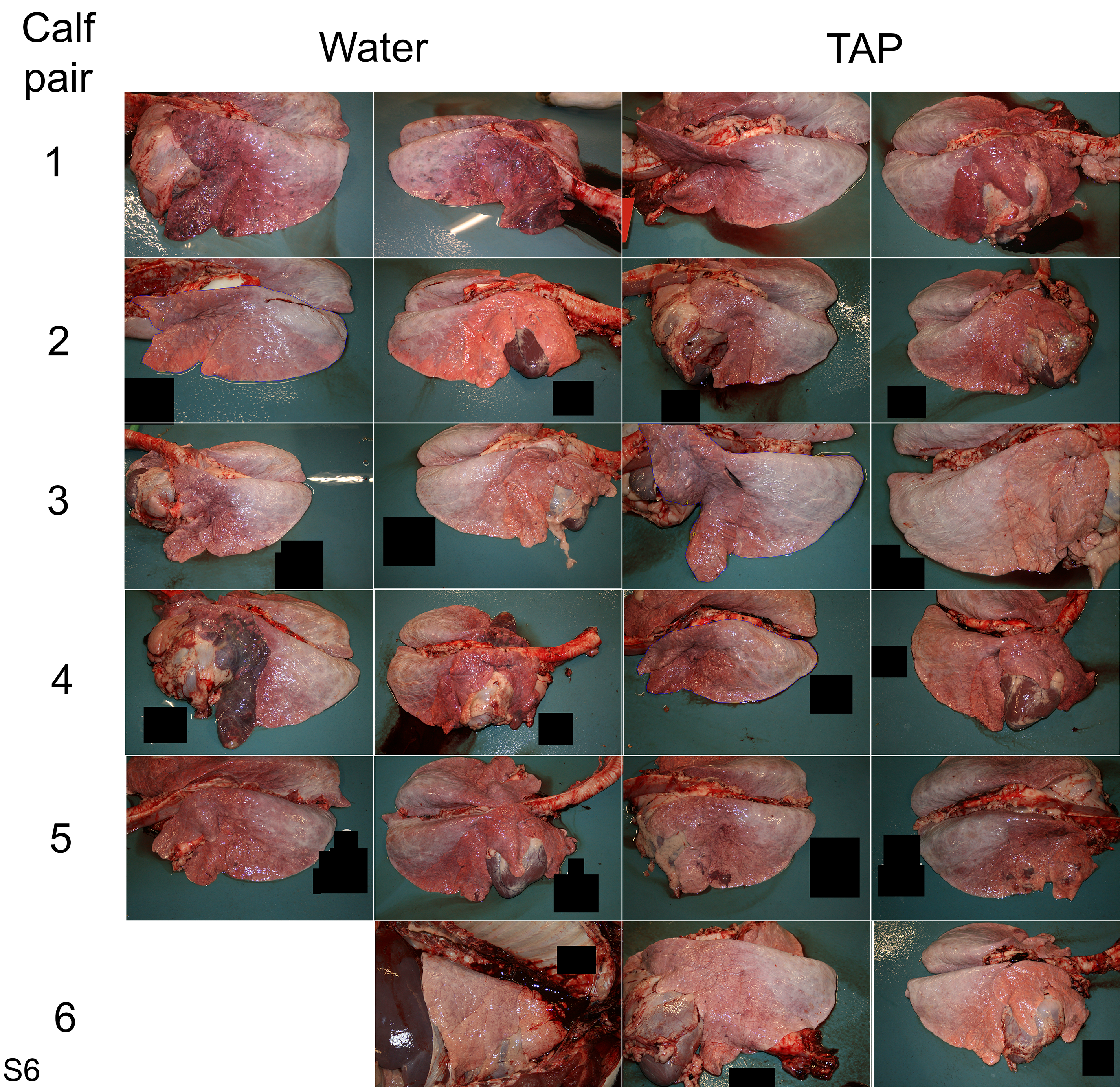

Supplement: S6 Fig — The images show the left and right lungs for the water-treated calf and the TAP-treated calf, for each calf pair in the study. Animal identification was blacked out for analysis. Image was missing for the left lung of water-treated calf #6 and analysis was based on the data collected at postmortem examination. (TIF) [file pone.0225533.s006.tif]

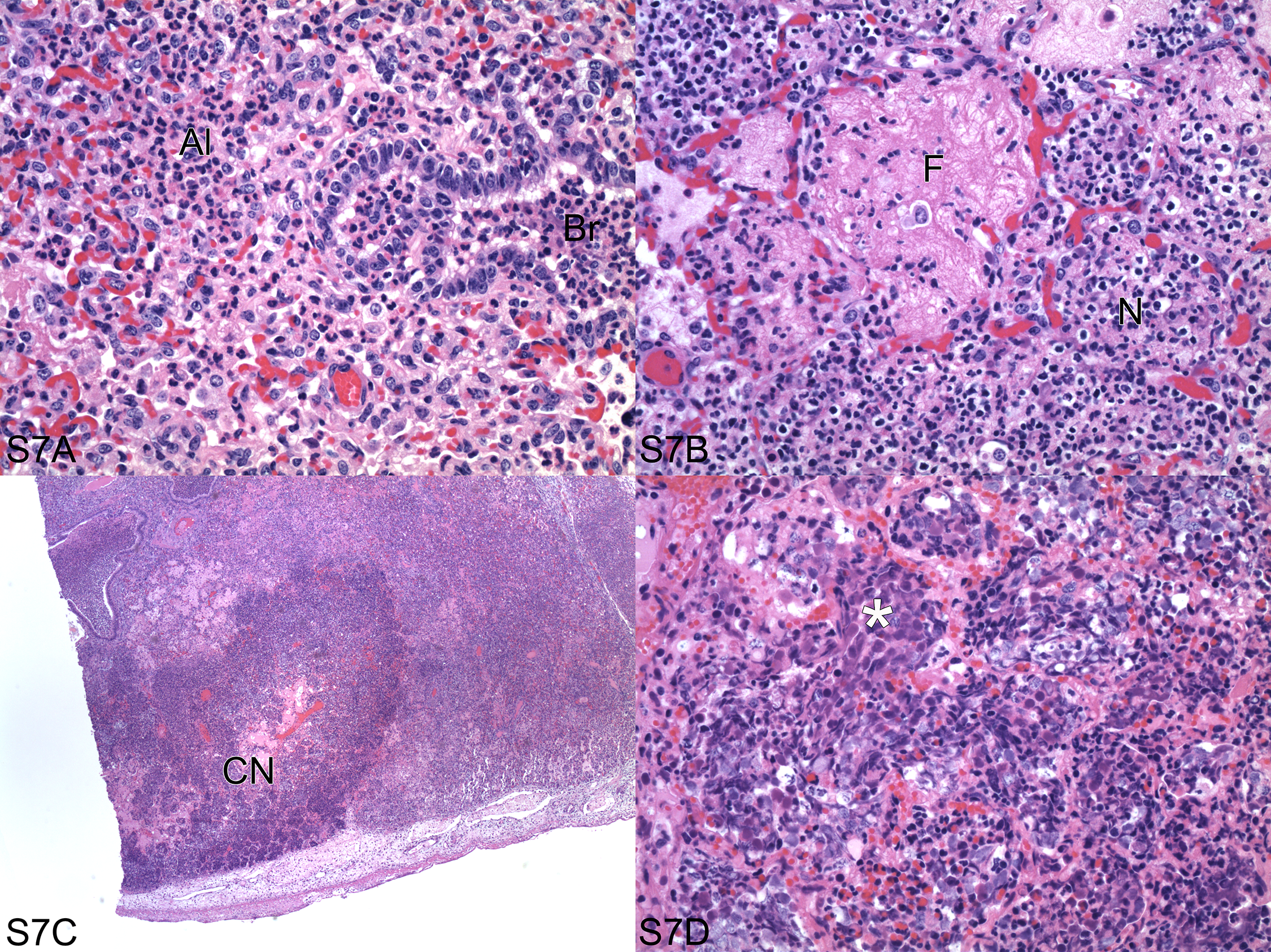

Supplement: S7 Fig — Calf pair #4, water-treated, 3 days after infection. (A) Well-preserved neutrophils fill a bronchiole (Br) and alveoli (Al). The alveoli are collapsed. (B) Alveoli are filled with fibrin (F) and necrotic neutrophils (N). (C) An inflamed lobule of lung contains a focal area of coagulation necrosis (CN) surrounded by a densely basophilic rim of leukocytes. (D) The densely basophilic rim shown in image C contains necrotic leukocytes that have streaming of their chromatin (*) typical of oat cells. (TIF) [file pone.0225533.s007.tif]

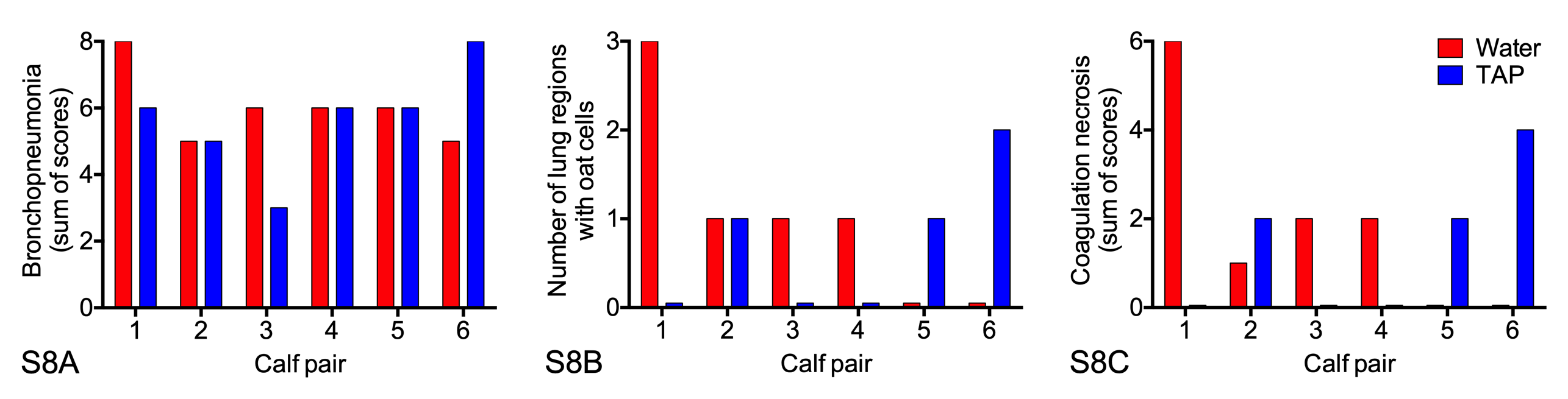

Supplement: S8 Fig — Grossly visible lesions were selectively sampled in three regions of lung: cranioventral, caudodorsal and caudoventral. Histologic lesions were semi-quantitatively scored based on bronchopneumonia (0–3), oat cells (0 or 2), and foci of coagulation necrosis (0–2). (A) The data show the sum of bronchopneumonia scores across the 3 sampled areas of lung. (B) The number of lung regions with oat cells (necrotic leukocytes with streaming chromatin). (C) The sum of coagulation necrosis scores across the 3 sampled areas of lung. (TIFF) [file pone.0225533.s008.tiff]

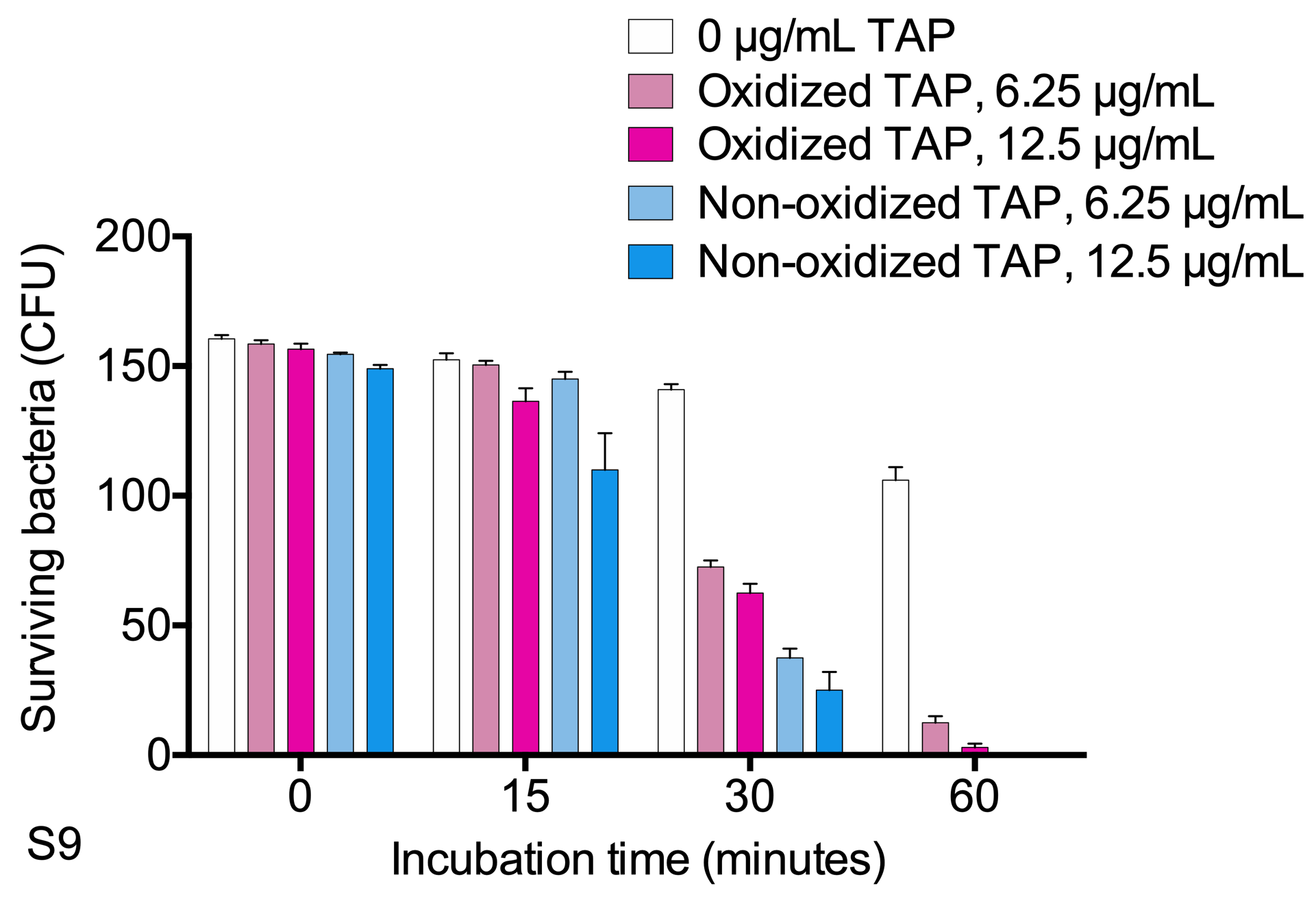

Supplement: S9 Fig — Different concentrations of oxidized or non-oxidized TAP or buffer were incubated with M. haemolytica. At 0, 15, 30 and 60 minutes, samples were inoculated onto blood agar plates, and the number of colonies (surviving bacteria) were counted the next day. CFU, colony-forming units. (TIFF) [file pone.0225533.s009.tiff]
