# Supplementary material for: Effect of tracheal antimicrobial peptide on the development of Mannheimia haemolytica pneumonia in cattle
Source: PLoS One. 2019 Nov 26;14(11):e0225533. doi: 10.1371/journal.pone.0225533 (PMC6879128; doi:10.1371/journal.pone.0225533)
Supplement: S1 Table — The clinical score was the sum of these components. The maximal clinical score was 17. (DOCX) [file pone.0225533.s010.docx]

## S1 Table. Clinical scoring scheme and humane endpoints.

The clinical score is the sum of the following components. The maximal clinical score is 17.

1. Demeanor: euthanize if 3 or greater.

• 0 = Not depressed

• 1 = Rarely stands alone, ears droop slightly, moves away when people approach

• 2 = Walks slowly, lethargic, sometimes stands with head low, easy to corner but difficult to catch

• 3 = Uninterested in environment, little response when people enter pen, easy to catch, lies in sternal recumbency frequently.

• 4 = Lies down most of the time, stands only occasionally, doesn't respond when people enter pen

2. Appetite (only assessed when food is newly available): euthanize if 3 on 2 consecutive feedings.

• 0 = Animal is seen eating with normal vigour

• 1 = Eats with less vigour than normal

• 2 = Suckles milk or nibbles food, but little is consumed

• 3 = Doesn't drink milk / doesn’t eat hay or grain

3. Strength: euthanize if 3 or greater.

• 0 = Normal and difficult to catch

• 1 = Walks slowly, mildly unsteady gait, easier to catch than normal

• 2 = Staggers or knuckles occasionally, recumbent less than 50% of the time, becomes recumbent from standing when people are in the pen, obviously unsteady gait

• 3 =Recumbent most of the time but will rise when stimulated.

• 4 = Recumbent and will not rise when stimulated

4. Effort of breathing: euthanize if 3 or greater.

• 0 =Normal breathing pattern. Mild but detectable increase in respiratory effort when stressed

• 1 = Obvious increase in respiratory effort when stressed, subtle increase when not stressed

• 2 = Obvious increase in respiratory effort when not stressed (observed from a distance)

• 3 =Open mouth breathing, marked increase in respiratory effort.

5. Cough.

• 0 = no cough

• 1 = cough elicited by gentle tracheal palpation

• 2 = infrequent spontaneous cough

• 3 = frequent spontaneous cough
